# Genome-wide association study implicates the role of *TBXAS1* in the pathogenesis of depressive symptoms among the Korean population

**DOI:** 10.1038/s41398-024-02777-3

**Published:** 2024-02-06

**Authors:** Kyungtaek Park, Ah Ra Do, Yuree Chung, Min Ji Kim, Sang Jin Rhee, Dae Hyun Yoon, Seung Ho Choi, Sung Joon Cho, Han-Na Kim, Yong Min Ahn, Sungho Won

**Affiliations:** 1 Institute of Health and Environment, Seoul National University, Seoul, Republic of Korea; 2 Interdisciplinary Program of Bioinformatics, College of Natural Sciences, Seoul National University, Seoul, Republic of Korea; 3 Department of Public Health Sciences, Graduate School of Public Health, Seoul National University, Seoul, Republic of Korea; 4 Department of Neuropsychiatry, Seoul National University Hospital, Seoul, Republic of Korea; 5 Department of Psychiatry, Seoul National University College of Medicine, Seoul, Republic of Korea; 6 Department of Psychiatry, Seoul National University Hospital Healthcare System Gangnam Center, Seoul, Republic of Korea; 7 Department of Internal Medicine, Healthcare Research Institute, Healthcare System Gangnam Center, Seoul National University Hospital, Seoul, Republic of Korea; 8 Department of Psychiatry, Kangbuk Samsung Hospital, Sungkyunkwan University School of Medicine, Seoul, Republic of Korea; 9 Workplace Mental Health Institute, Kangbuk Samsung Hospital, Seoul, Republic of Korea; 10 Department of Clinical Research Design and Evaluation, Samsung Advanced Institute for Health Sciences and Technology, Sungkyunkwan University, Seoul, Republic of Korea; 11 Biomedical Statistics Center, Research Institute for Future Medicine, Samsung Medical Center, Seoul, Republic of Korea; 12 Institute of Human Behavioral Medicine, Seoul National University Medical Research Center, Seoul, Republic of Korea

**Keywords:** Depression, Comparative genomics

## Abstract

Although depression is an emerging disorder affecting many people worldwide, most genetic studies have been performed in European descent populations. Herein, a genome-wide association study (GWAS) was conducted in Korean population to elucidate the genomic loci associated with depressive symptoms. Two independent cohorts were used as discovery datasets, which consisted of 6474 (1484 cases and 4990 controls) and 1654 (557 cases and 1097 controls) Korean participants, respectively. The participants were divided into case and control groups based on the Beck Depression Inventory (BDI). Meta-analysis using the two cohorts revealed that rs6945590 was significantly associated with the risk of depressive symptoms [*P* = 2.83 × 10^−8^; odds ratio (OR) = 1.23; 95% confidence interval (CI): 1.15–1.33]. This association was validated in other independent cohorts which were another Korean cohort (258 cases and 1757 controls) and the East Asian study of the Psychiatric Genomics Consortium (PGC) (12,455 cases and 85,548 controls). The predicted expression levels of *thromboxane A synthase 1* gene (*TBXAS1*), which encodes the enzyme thromboxane A synthase 1 and participates in the arachidonic acid (AA) cascade, was significantly decreased in the whole blood tissues of the participants with depressive symptoms. Furthermore, Mendelian randomization (MR) analysis showed a causal association between *TBXAS1* expression and the risk of depressive symptoms. In conclusion, as the number of risk alleles (A) of rs6945590 increased, *TBXAS1* expression decreased, which subsequently caused an increase in the risk of depressive symptoms.

## Introduction

Depression is a common mental illness with symptoms, including sadness, lack of satisfaction, and sleep disturbance. Worldwide, approximately 280 million people suffer from depression in 2019, and the number only seems to increase from 1990 to 2019 [1]. The prevalence of depression in South Korea also has increased annually [2]. In particular, considering that depression is a major risk factor for suicide [3], the disease has been a big burden on South Korea. In 2019, the suicide rate recorded in South Korea was 24.6 per 100,000, which is the highest among the Organization for Economic Co-operation and Development countries [4].

Several loci associated with the risk of depression have been identified with efforts from many participants and researchers. For example, a meta-analysis with the highest number of participants to date showed that 102 loci are susceptible to the disease [5]. However, as the majority of genome-wide association study (GWAS) participants are of European descent [6], most depression studies have focused on the European populations [7]. Although a few independent variants have been reported in East Asian populations [8, 9], more studies are needed, especially in non-European populations, to fully understand the etiology of the disease.

Another difficulty in shedding light on this disorder is that it is a polygenic trait; that is, its individual variants have only a small effect, which requires a large sample size to discover the susceptible loci. To overcome this problem, many GWASs have been performed comprising participants with self-reported depression [5, 9–12], which is supported by the fact that self-declared depression and clinically diagnosed major depressive disorder (MDD) show a strong genetic correlation [11, 13].

Here, we carried out GWAS to uncover variants associated with the risk of depressive symptoms in Korean population. Approximately 12,000 participants from three independent cohorts belonging to two different hospitals visited the institutes for a general health check-up. Moreover, their depression phenotypes were self-rated using Beck Depression Inventory (BDI) or Center for Epidemiologic Studies-Depression Scale (CES-D). Following GWAS, Mendelian randomization (MR) study was performed to identify the causal genomic loci.

## Subjects and methods

### Health and prevention enhancement (H-PEACE) and gene-environment of interaction and phenotype (GENIE) cohorts

In the study of discovery stage, the H-PEACE and GENIE cohorts were used. They consisted of participants who visited the Seoul National University Hospital Healthcare System Gangnam Center for health check-ups from 2003 to 2017 [14]. Among them, 7999 and 2349 participants from the H-PEACE and GENIE cohorts, respectively, were genotyped on 833,000 variants using the Affymetrix Axiom Korean Chip (v1.0) [15]. The variant calling was performed using the K-Medoid clustering method [16].

### Beck depression inventory

The degree of depression of the participants in the two cohorts was measured using original BDI. It is one of the most widely used self-scored measures of depression, comprising 21 items to evaluate how the participants had been feeling for the last two weeks [17]. Each item was rated on a four-point ordinal scale from 0 to 3, where a higher score represents a higher level of depression-related feelings. The total sum of the scores of 21 items was used as a criterion for depression.

In the cohorts, the BDI scores of a few participants had errors, because they had selected more than one point for an item or their responses for some items were missing. We regarded them as missing values and selected subjects who had missing values lower than 10.5, half of the total number of items. The average missing rates for each item in the H-PEACE and GENIE cohorts were 7.23% and 19.09%, respectively. We imputed missing values of the remaining participants by referring to (if any) their own scores measured during their check-ups at other times or scores of other participants who were close to them based on scores of other non-missing items using the missForest (v.1.4) R package [18]. In accordance with Beck et al. [19], we regarded participants whose sum of the imputed scores was lower than 10 as controls and the others as cases. In the case of participants who had multiple health check-ups, the result was extracted when they had the highest BDI score (if they had the same scores, the latest result was extracted).

### Genome-wide association study

After sample selection based on the BDI score, the genotype data were cleaned using the quality control steps suggested by Anderson et al. [20]. Participants whose genotype missing rate was higher than 0.03, estimated heterozygosity rate differed from the sample mean of all participants by more than three standard deviations, and the estimated pairwise identity-by-descent was higher than 0.185 with a higher genotype missing rate than that of their counterparts were excluded. Variants whose genotype missing rate was significantly different between the case and control by Fisher’s exact test (*P* < 1 × 10^−5^) and higher than 0.03, minor allele frequency (MAF) was lower than 0.05, and *P* value of the Hardy–Weinberg equilibrium (HWE) test was lower than 1 × 10^−3^ were excluded (Supplementary Fig. 1).

Subsequently, the genotype data were imputed using the Michigan Imputation Server (v.1.1) [21]. Haplotype Reference Consortium r1.1 2016 and other/mixed population were chosen as reference panels, and phasing and imputation were performed using Eagle v2.4 and Minimac4, respectively. Among the imputed genotypes, variants whose MAF was higher than 0.05, genotype missing rate was lower than 0.03, HWE test was not significant at *P* value 1 × 10^−3^, and imputation quality measure “INFO” was higher than 0.8 were extracted (Supplementary Fig. 1).

Further, the GWAS was performed in each cohort after adjusting for the effects of sex, age, body mass index (BMI), and the top four principal component (PC) scores using plink (v1.90b3.44) [22]. For 67 and 18 (approximately 1%) participants in the H-PEACE and GENIE cohorts, respectively, BMIs were not observed and were imputed using missForest by referring to those of other participants who had similar sex and age [18]. The PC scores were calculated using pruned genotyped variants extracted by using *--indep-pairwise 50 5 0.2* option of plink. Meta-analysis was also performed with weights of inverse of the corresponding standard errors of each GWAS result using METAL (v2011-03-25) [23]. Cochran’s Q and I square statistics were used to test whether variants displayed heterogeneity across the different cohorts.

For genomic data analysis, in addition to plink (v1.90b3.44) and METAL (v2011-03-25), ONETOOL (v1.0) [24] and R (v3.6.3) were utilized.

Moreover, LocusZoom (http://locuszoom.org/) was used to generate a regional plot of ±0.5 Mb flanking region of a significant variant [25]. Genome build and linkage disequilibrium (LD) population were selected as hg19 and 1000 Genomes Nov 2014 ASN, respectively. The other options were not manipulated.

### Replication cohorts

For validation, Kangbuk Samsung Cohort Study (KSCS) cohort was used. It is a prospective cohort study to evaluate the natural history, prognosis, and genetic and environmental determinants of a wide range of health traits and diseases among Korean adults. 2011 participants were genotyped using the Illumina HumanCore BeadChips 12 v. Depressive symptoms and severity were assessed using CES-D [26]. For identifying individuals at risk for clinical depressive symptoms, it uses 16 or above as cut-off score [27]. We divided the participants into two groups based on the cut-off score: depressed and non-depressed groups. The analysis steps used were the same as in H-PEACE and GENIE, except that 0.05 was used as the genotype missing rate cutoff in the participant QC step, and 0.02, 0.01, and 1 × 10^−6^ were used as the genotype missing rate, MAF, and HWE cutoffs, respectively, in the variant QC step. Furthermore, an older version of Minimac, Minimac3, was used in the imputation step.

Moreover, we referred an independent study of which meta-analysis of GWASs on depression in nine cohorts of East Asian ancestry [9]. The summary statistics, excluding the three cohorts, WHI, 23andME, and UKB, were downloaded from https://www.med.unc.edu/pgc/download-results/.

### Expression quantitative trait loci analysis

LDexpress [28], a web-based tool implemented in LDlink [29] (https://analysistools.cancer.gov/LDlink/), was used to search for genes whose expression levels were associated with a significant variant from the GWAS results. In the East Asian population, genes in several tissues were identified, the expression levels of which were significantly associated (*P* < 1 × 10^−5^) with variants which were highly correlated (*R*^2^ > 0.8) with a significant variant in ±1 Mb window. The correlation between variants was estimated in the East Asian population using LDpair implemented in LDlink.

### Transcriptome-wide association study

Gene expression levels in the whole blood tissues of the participants were predicted using PrediXcan [30]. The imputed genomic data of each cohort were used as input for the analysis. The gene-wise association between gene expression (a response variable) and self-rated depression (an explanatory variable) was estimated using a linear regression model with the same covariates used in the GWAS. Moreover, the false discovery rate (FDR) was estimated for each chromosome.

### MR analysis

In this study, MR analysis was performed to identify the causal relationship between a gene and the risk of depressive symptoms. Generalized summary-data-based MR (GSMR) [31] implemented in Genome-wide complex trait analysis (GCTA) (v1.94.0b) [32] was used for the analysis with its default options. Additionally, to corroborate the MR result, we employed several methods, including the median method, inverse-variance weighted (IVW) method, MR-Egger, and MR-PRESSO [33, 34]. MendelianRandomization R package (v0.7.0) was used for implementing the median, IVW, and MR-Egger methods [35]. We used default options for these methods, with exception of specifying the number of iterations as 10,000. For the MR-PRESSO analysis, we utilized MRPRESSO R package (v1.0) with following options: *OUTLIERtest* = *T, DISTORTIONtest* = *T, NbDistribution* = *10000, and SignifThreshold* = *0.05*. These methods required two summary statistics from regression models between variants and exposure or outcome and reference samples for LD estimation.

We estimated the summary statistic of the association between the expression levels of genes and variants in the imputed H-PEACE dataset using a linear regression model with the same covariates used in the GWAS. The GWAS result of the imputed GENIE dataset was used for the other summary statistic. East Asian samples in 1000 Genomes Phase 3 v5a were used as reference samples.

In order to select instrumental variables (IVs) that satisfy the assumptions of MR, we first identified variants that were independently and significantly associated with the exposure (R^2^ < 0.05; *P* < 5 × 10^−8^). To exclude those with pleiotropic effect, we further pruned these variants using the HEIDI method as implemented in GSMR. IVs displaying pleiotropic effects, as estimated by the MR-PRESSO, were also eliminated. In addition, we tested for heterogeneity and weak instrumental bias using the *Q*_*GX*_ and ${I}_{{GX}}^{2}$ [36].

### Association study between variants or a gene and each depressive symptom

GWASs of each item were performed considering the phenotype as 0 if the score was 0 or 1 otherwise and adjusting the effects of sex, age, BMI, and top four PC scores in each cohort. Meta-analyses for each item were performed using METAL. Associations between gene expression and the phenotype of each item were estimated with the same model used in the GWAS, except for replacing the number of minor alleles of a variant with the expression level of a gene in the two cohorts. Further, their *Z* statistics were summed by weighting their corresponding inverses of standard errors, and the *P* values were calculated using the weighted Z statistics. Statistical analyses were performed using R and Rex (v3.6.1) [37].

### Estimation of phenotypic and genotype correlations between depressive symptoms

Based on the scores (0–3 scale), pairwise phenotypic correlations were estimated between the 21 BDI items in the H-PEACE and GENIE cohorts, respectively. Moreover, the items in combining the two cohorts were grouped into four clusters using *hcluster* function implemented in the amap R package (v0.8-18) with designating method and link options as Pearson and average, respectively. The phenotypes in each cluster were divided into two groups based on a cutoff in proportion to the number of items in the cluster. For example, the cutoff was set to 6.67, which was 10 times 14 divided by 21, in cluster 4 which included 14 items. GWASs were performed in each cluster of the two cohorts, respectively, with adjusting the effects of the same covariates used as above. Then, the results were combined by METAL.

In each cohort, genetic correlation between items were estimated by performing a bivariate GREML analysis [38] implemented in GCTA with adjusting the effects of sex, age, BMI, and top four PC scores, respectively. Prevalence of each item was estimated as ratio of non-zero score of the item and specified in the GREML analysis. Genetic relationship matrix was calculated by pruned variants with having larger than 0.1 MAF.

### Ethics statement

This study was approved by the institutional review boards of Seoul National University Hospital (H-1708-120-880) and Kangbuk Samsung Hospital (2019-08-006). All participants were provided written informed consent and the study was performed according to relevant guidelines and regulations.

## Results

### GWAS reveals that rs6945590 is associated with the risk of depressive symptoms

We performed a GWAS to elucidate the genetic loci susceptible to the risk of depressive symptoms in H-PEACE (4,233,391 variants in 1484 cases and 4990 controls) and GENIE (4,285,695 variants in 557 cases and 1097 controls) cohorts (Table 1). The proportion of females was higher in the cases than in the controls in both cohorts. Moreover, the age and BMI were similar between the cases and controls in both cohorts. Although several putative loci (*P* < 1 × 10^−5^) were identified, there were no significant variants at the conventional significance level (*P* < 5 × 10^−8^) in both cohorts (Supplementary Fig. 2 and Supplementary Tables 1, 2). The most significant variants in the H-PEACE and GENIE cohorts were rs745327 (chr12:122456524) and rs16826069 (chr1:39797055), respectively.

**Table 1:** Participant characteristics and the number of variants in the H-PEACE and GENIE cohorts.

	H-PEACE		GENIE	
	Case (*N* = 1484)	Control (*N* = 4990)	Case (*N* = 557)	Control (*N* = 1097)
Age, mean (SD), yr	49.6 (10.9)	49.6 (9.7)	50.2 (10.1)	50.2 (9.3)
Women, N (%)	864 (58.2)	1829 (36.7)	262 (47.0)	338 (30.8)
BMI, mean (SD), kg/m^2*^	22.8 (3.2)	23.2 (2.9)	23.4 (3.2)	23.7 (2.9)
Variants after imputation, N	4,233,391		4,285,695	

**Fig. 1:**
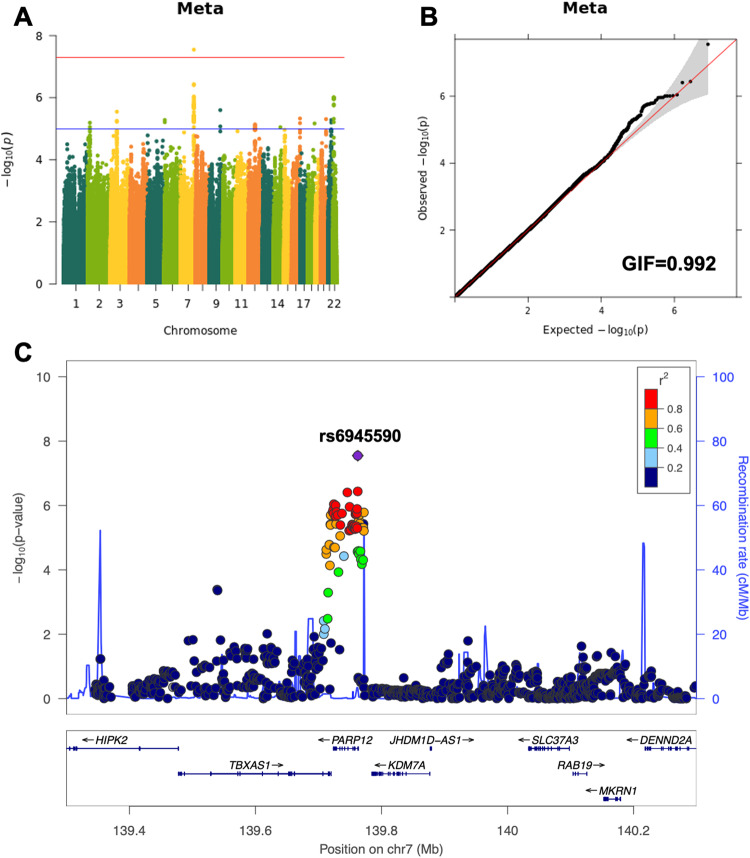
Meta-analysis results of the risk of depressive symptoms. **A** Manhattan plot. Red and blue lines represent significance level 5 × 10^−8^ and 1 × 10^−5^, respectively. **B** Quantile–quantile plot. Gray region indicates point-wise 95% confidence interval (CI) of *P* values. **C** Regional plot. Purple square represents rs6945590 and circles represent other variants. Colors represent degree of correlation (r^2^) with rs6945590, and genes near the variant are shown below. p, *P* value; GIF, genomic inflation factor; chr, chromosome.

Subsequently, a meta-analysis was performed using both cohorts. We analyzed a total of 4,141,113 variants in 8128 participants (2041 cases and 5087 controls), of which one novel locus was identified (Fig. 1). At the locus, rs6945590 (chr7:139762282; 7q34) was significant (*P* = 2.83 × 10^−8^) at the conventional significance level (Fig. 1A). Moreover, the result was found to be reliable considering its quantile-quantile plot and a genomic inflation factor of 0.992 (Fig. 1B). *P* values of the variants that were highly correlated with rs6945590 were lower than those of the variants that were not (Fig. 1C). In addition, rs6945590 is located in the intron region of the *poly(ADP-ribose) polymerase family member 12 (PARP12)* gene according to dbSNP (www.ncbi.nlm.nih.gov/snp). Moreover, several genes, such as the *thromboxane A synthase 1* gene *(TBXAS1)* are in close proximity to this gene (Fig. 1C).

Details regarding the GWAS results for rs6945590 are shown in Table 2. The risk and protective alleles of the variant were A and G, respectively. Moreover, the odds ratios (*ORs*) of the risk allele were 1.22 (95% confidence interval (CI): 1.12–1.33) and 1.26 (95% CI: 1.09–1.46) in H-PEACE and GENIE cohorts, respectively. The meta-analysis of both cohorts showed an OR of 1.23 (95% CI: 1.15–1.33). In the meta-analysis, which only included 5905 participants (1212 cases and 4693 controls) without any missing values for all BDI items, the OR for rs6945590 was found to be 1.22 (95% CI: 1.11–1.34) with a *P* value of 3.06 × 10^−5^. The risk allele frequencies (RAFs) of the cases were 0.608 and 0.584 in H-PEACE and GENIE cohorts, respectively. Moreover, the RAFs of the controls were 0.568 and 0.544 in H-PEACE and GENIE cohorts, respectively. Furthermore, these RAF values were found to be similar to those of the Korean population retrieved from Korean Reference Genome Database (KRGDB) (0.550) [39] and gnomAD (0.566) [40].

**Table 2:** Results of GWA studies and meta-analysis for rs6945590.

Variants	Chr	BP	Risk/Protective alleles	H-PEACE			GENIE			Meta-analysis of H-PEACE and GENIE		KSCS			Meta-analysis of H-PEACE, GENIE, and KSCS	
				*P* value	OR (95% CI)	RAF (Case/Control)	*P* value	OR (95% CI)	RAF (Case/Control)	*P* value	OR (95% CI)	*P* value	OR (95% CI)	RAF (Case/Control)	*P* value	OR (95% CI)
rs6945590	7	139762282	A/G	3.79 × 10^−6^	1.22 (1.12–1.33)	0.608/0.568	1.87 × 10^−3^	1.26 (1.09–1.46)	0.584/0.544	2.83 × 10^−8^	1.23 (1.15–1.33)	1.64 × 10^−2^	1.28 (1.05–1.56)	0.409/0.360	1.59 × 10^−9^	1.24 (1.16–1.33)

*Chr* chromosome, *BP* base pair, *OR* odds ratio, *CI* confidence interval, *RAF* risk allele frequency.

We analyzed whether rs6945590 is associated with the risk of depressive symptoms or depression in other independent cohorts. In the KSCS cohort (4,792,372 variants in 258 cases and 1752 controls), this association was validated at a nominal *P* value 0.05 (*P* = 1.64 × 10^−2^; *OR* = 1.28; 95% *CI*: 1.05–1.56) (Table 2). When the meta-analysis of the H-PEACE, GENIE, and KSCS cohorts was performed, the association became more significant (*P* = 1.59 × 10^−9^; *OR* = 1.24; 95% *CI*: 1.16–1.33). Additionally, the results of rs6945590 indicates the little evidence of heterogeneity across the three cohorts (*Q* = 0.212; *I*^2^ = 0). Moreover, according to the other study of depression in the East Asian population (7,440,922 variants in 12,455 cases and 85,548 controls) [9], rs6945590 was significantly associated with the risk of depression at a nominal *P* value 0.05 (*P* = 2.28 × 10^−2^; *OR* = 1.04; 95% *CI*: 1.01–1.07).

In GWAS Catalog (www.ebi.ac.uk/gwas), traits that significantly associated with rs6945590 were not found, and in LDtrait (ldlink.nci.nih.gov/?tab=ldtrait), a web-based tool implemented in LDlink, association also was not reported between depression risk and rs6945590 or its highly correlated variants (*R*^2^ > 0.8), however, some traits such as white blood cell count, monocyte count, and myocardial infarction had significant association with highly correlated variants with rs6945590 (*P* < 5 × 10^−8^; Supplementary Table 3).

### *TBXAS1* expression level is associated with rs6945590 and the risk of depressive symptoms

Genes for which rs6945590 was an eQTL were explored using LDexpress in the East Asian population. rs6945590 was found to be associated with *PARP12* expression in tibial nerve (*P* = 2 × 10^−6^) and testis (*P* = 6 × 10^−6^) tissues (Supplementary Table 4). Among pairs between genes and variants in the LD relationship with rs6945590 (*R*^2^ > 0.8), *TBXAS1* and rs10242990 (chr7:139730500) pair in the whole blood tissue was the most significantly associated (*P* = 9 × 10^−15^; Supplementary Tables 5, 6). rs10242990 and rs6945590 were highly correlated (*R*^2^ = 0.807) and the risk allele (A) of rs6945590 was positively associated with the effect allele (G) of rs10242990. As the number of effect alleles of rs10242990 increased, *TBXAS1* expression levels decreased in the whole blood tissue (*β* = –0.098; Supplementary Table 5). *PARP12* also had eQTL variants in the LD region of rs6945590, but its expression levels were not significantly associated with the variants in the whole blood or brain tissues at a nominal *P* value 1 × 10^−5^ (Supplementary Table 6).

**Fig. 2:**
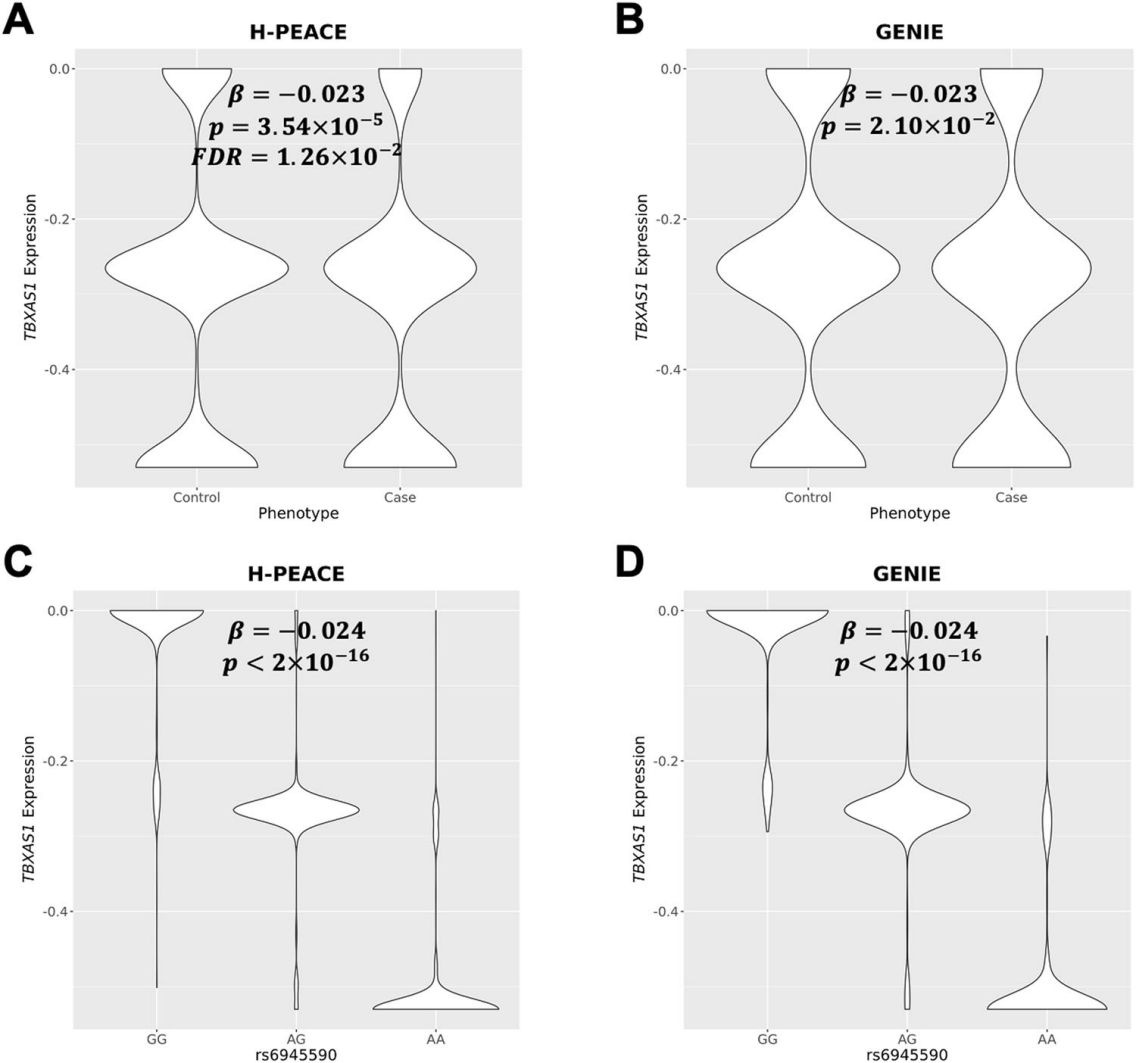
Association between *TBXAS1* expression levels and risk of depressive symptoms or rs6945590 in whole blood. Violin plots of predicted *TBXAS1* expression levels according to the depression phenotypes of the (**A**) health and prevention enhancement (H-PEACE) and (**B**) gene-environment of interaction and phenotype (GENIE) cohorts and alleles of rs6945590 of (**C**) H-PEACE and (**D**) GENIE cohorts. *β*, estimate of the association between *TBXAS1* expression and rs6945590 or the phenotypes; p, *P* value; FDR, false discovery rate.

We predicted the expression levels of genes in the whole blood tissue of the participants from the genotypes of the H-PEACE and GENIE cohorts using PrediXcan to identify the genes associated with the risk of depressive symptoms (Fig. 2). In the H-PEACE cohort, the predicted *TBXAS1* expression level was negatively significantly associated with the risk of depressive symptoms at an FDR of 0.05 (*β* = –0.023; *P* = 3.54 × 10^−5^; FDR = 1.26 × 10^−2^) (Fig. 2A), and the association was validated in the GENIE cohort at a nominal *P* value 0.05 (*β* = –0.023; *P* = 2.10 × 10^−2^) (Fig. 2B). *TBXAS1* expression level was also significantly associated with rs6945590 in both cohorts. As the number of risk alleles (A) of rs6945590 increased, *TBXAS1* expression decreased (Fig. 2C, D).

Moreover, *lanosterol synthase* (*LSS*) and *YbeY metalloendoribonuclease* (*YBEY*) may be other putative genes associated with the risk of depressive symptoms (Supplementary Fig. 3). *LSS* was predicted to be significantly more expressed in the cases than in the controls in the H-PEACE cohort (*β* = 0.046; *P* = 1.43 × 10^−4^; FDR = 6.30 × 10^−3^) and the same pattern was observed in the GENIE cohort (*β* = 0.033; one tail *P* = 4.28 × 10^−2^) (Supplementary Fig. 3A, B; and Supplementary Table 7). The predicted *YBEY* expression levels significantly decreased in the cases compared to the controls in the H-PEACE cohort at an FDR of 0.1 (*β* = –0.031; *P* = 3.40 × 10^−3^; FDR = 7.48 × 10^–2^), and it was validated in the GENIE cohort (*β* = –0.038; *P* = 4.92 × 10^−2^) (Supplementary Fig. 3C, D; and Supplementary Table 7). Although the genes ENSG00000235878, *4-hydroxyphenylpyruvate dioxygenase* (*HPD*), *cysteine and tyrosine rich 1* (*CYYR1*), and *spermatogenesis and centriole associated 1 like* (*SPATC1L*) showed a significant association with the risk of depressive symptoms in the H-PEACE cohort, they did not in the GENIE cohort (Supplementary Table 7).

### MR study shows that *TBXAS1* may be a causal gene of depressive symptoms

**Fig. 3:**
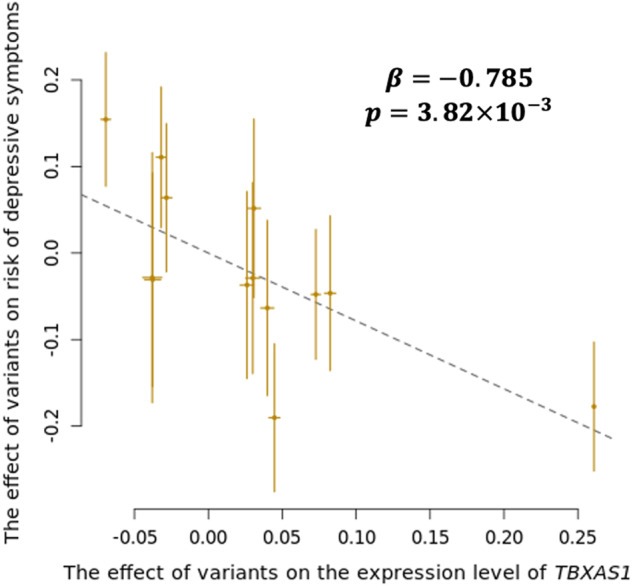
Causal effect of *TBXAS1* expression on the risk of depressive symptoms. Effects of 13 instrumental variables (IVs) are shown in yellow dots with their ±1 standard error (yellow lines). Slope of dashed lines represents the causal effect of *TBXAS1* expression on the risk of depressive symptoms. *β*, estimate of the causal effect; p, *P* value.

We performed a two-sample MR study using the GSMR to test whether genes and the risk of depressive symptoms had a causal relationship. The two genome-wide association results were from the analyses: (1) between variants and *TBXAS1* expression in the H-PEACE cohort and (2) between variants and the risk of depressive symptoms in the GENIE cohort. With 13 independent IVs (R^2^ < 0.05; *P* < 5 × 10^−8^), the study revealed that a decrease in *TBXAS1* expression led to an increase in the risk of depressive symptoms (*β* = –0.785; *P* = 3.82 × 10^−3^) (Fig. 3). The causal relationship was further substantiated when analyzed using other MR methods (Supplementary Table 8). Furthermore, there was no significant results of the variants being heterogeneous or having a weak instrumental bias (*Q*_*GX*_ = 6.0729; *P* value of *Q*_*GX*_ = 0.8685; ${I}_{{GX}}^{2}=1$). When the *P* value cutoff for IV selection was increased to 1 × 10^−5^, the effect was still significant, with 23 IVs (*β* = –0.783; *P* = 3.42 × 10^−3^) (Supplementary Fig. 4A). The reverse causation could not be tested because there were not enough variants available as IVs even when the cutoff was set to 1 × 10^−5^.

The causal relationships between *LSS* or *YBEY* and the risk of depressive symptoms were also tested. Although there were not enough IVs when the cutoff was set to the conventional threshold, 5 × 10^−8^, for both directions, *LSS* may be a putative causal gene for depressive symptoms considering the result when the cutoff was 1 × 10^−5^ with 17 IVs (*β* = 0.280; *P* = 3.51 × 10^−2^) (Supplementary Fig. 4B). If *LSS* expression increases, the risk of depressive symptoms may increase. *YBEY* may also have a causal relationship with the risk of depressive symptoms. A decrease in its expression appeared to increase the risk of depressive symptoms, but its nominal *P* value was slightly higher than 0.05 (*β* = –0.265; *P* = 6.99 × 10^−2^). It was estimated with 10 IVs that were selected at the conventional cutoff (Supplementary Fig. 4C). There were not enough variants available as IVs to test the reverse causation even when the cutoff was 1 × 10^−5^ in both genes.

### Some symptoms of depression are more highly associated with rs6945590 than others

**Fig. 4:**
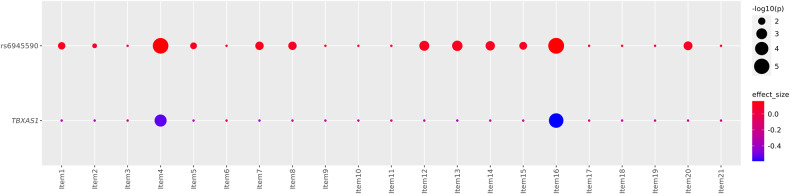
Associations between each item and rs6945590 or *TBXAS1*. Effect sizes are represented by circles with colors ranging from red (positive) to blue (negative). Size of the circle represents *P* values expressed as -log10 scale. Circles with the smallest size indicate that the association is not significant. 12 and 2 out of the 21 items were significantly associated with rs6945590 and *TBXAS1*, respectively. p, *P* value.

The associations between rs6945590 or *TBXAS1* expression and each item were investigated. As the number of risk alleles of rs6945590 increased, the ratios of non-zero value of 12 out of the 21 items (items 1, 2, 4, 5, 7, 8, 12, 13, 14, 15, 16, and 20) significantly increased at an FDR of 0.05 in (Fig. 4). Among them, items 4 (lack of satisfaction) and 16 (sleep disturbance) had the smallest *P* values. *TBXAS1* expression was significantly associated with the items 4 and 16 at an FDR of 0.05 (Fig. 4).

Scores for each item were significantly correlated with each other except for item 19 (weight loss). Moreover, the scores for items 9 (suicide) and 10 (cry) showed the highest correlation in both the H-PEACE and GENIE cohorts (Supplementary Fig. 5A). We clustered the items based on their pairwise score correlations (Supplementary Fig. 5B). There were four clusters: cluster 1 included item 19; cluster 2 included item 6; cluster 3 included items 1, 2, 3, 9, and 10; cluster 4 included the other remaining 14 items. 10 of 12 items which had significant association with rs6945590 were included in the cluster 4.

GWASs of each cluster showed that rs6945590 was significantly associated with the risk of depressive symptoms in only cluster 4 (*P* = 1.95 × 10^−8^; *OR* = 1.22; 95% *CI*: 1.14–1.31; Supplementary Table 9). Moreover, there was no other significant variant in the four clusters (Supplementary Fig. 6).

Pairwise genetic correlations between items in each cohort were not significantly different from 0 at an FDR of 0.05 except items 5 (guilt) and 7 (self-hate) in GENIE cohort which was not significant in H-PEACE cohort (Supplementary Fig. 5C). Furthermore, GWASs were performed for each item using the same procedure as above. However, there was no significant variant at the 5 × 10^−8^ significance level (Supplementary Fig. 7).

## Discussion

In this study, rs6945590 was identified as a novel susceptible variant of depressive symptoms in the Korean population (Fig. 1, Table 2). The variant was shown to be significantly associated with the risk of depressive symptoms or depression in three independent cohorts consisting of Korean and one cohort consisting of East Asian. As the number of risk alleles of rs6945590 increased, the expression level of *TBXAS1* decreased (Fig. 2), subsequently increasing the risk of depressive symptoms (Fig. 3). Among symptoms of depression, lack of satisfaction and sleep disturbance were highly associated with rs6945590 and *TBXAS1* (Fig. 4).

Other than rs6945590, no variants were significantly associated with risk of depressive symptoms in our study including variants which were shown to be in European study [5]. Although 59 out of 102 significant variants in the European study existed in our dataset, only 2 variants (rs113188507 and rs9592461) had the same direction of OR with significant association with risk of depressive symptoms at nominal *P* value 0.05 in our study (Supplementary Table 10). It may implicate importance of our study to fill missing heritability of depression.

Patients with depression have not only heterogeneous symptoms, but also different drug responses. Only a third of the patients are treated by the currently developed drugs that largely target the monoamine pathway; the other half are partly cured by the drugs, and the rest do not respond to them [41, 42]. One promising remedy for this unmet need is to utilize the relationship between inflammation and neuropsychiatric diseases. In recent years, emerging evidence has shown that immune mechanisms can contribute to depression [43, 44]. For example, higher interleukin-6 levels in blood precede depression [45, 46] and anti-inflammatory treatments such as non-steroidal anti-inflammatory drugs (NSAIDs) show antidepressant effects [47].

The arachidonic acid (AA) cascade is an immune response pathway that plays a key role in many inflammatory diseases such as asthma [48]. AA is converted to prostaglandin H2 (PGH2), catalyzed by cyclooxygenase (COX), and subsequently to prostaglandins or thromboxane A2 (TXA2). In the latter step, thromboxane A synthase 1, encoded by *TBXAS1*, catalyzes the conversion of PGH2 to TXA2, which is known as an inducer of platelet aggregation.

Considering that TXA2 activates the immune response, which may be positively associated with the risk of depression, it appears that the risk of depressive symptoms may decrease with the decrease in *TBXAS1* expression. However, in the AA to TXA2 cascade, TXA2 itself may have a limited effect on depressive symptoms. However, the amount of AA appears to have an important effect on depression. Several studies have shown that the ratio of AA to long-chain omega-3 fatty acids is elevated in depression [49, 50]. Moreover, continued use of non-aspirin NSAIDs or high-dose aspirin (COX inhibitors that block the conversion of AA to PGH2) increases the incidence rate of depression [51]. Moreover, aspirin aggravates depressive symptoms [52, 53].

In addition to COX, AA is metabolized by lipoxygenases (LOXs). LOXs catalyze the conversion of AA to leukotriene, which has broad proinflammatory effects [54]. For example, knockdown of the leukotriene receptor prevents depressive behavior in mice [55]. Moreover, leukotriene receptor antagonists were reported to be associated with sleep disorder [56, 57]. Leukotriene is mainly synthesized by leukocytes and monoblastic lineages such as monocytes [58]. In addition, according to the Human Protein Atlas (https://www.proteinatlas.org/), *TBXAS1* is more highly expressed in monocytes than in other cell types in peripheral blood mononuclear cells.

LDtrait showed that rs6945590 was highly correlated with the variants that were significantly associated with the immune cell counts in the East Asian population (Supplementary Table 3). rs2269996 (chr7:139724358) was significantly associated with the white blood cell and monocyte counts. rs7781964 (chr7:139749346) was significantly associated with the lymphocyte count. Moreover, studies have shown that white blood cell count and monocyte count are positively associated with depression risk [59, 60].

Therefore, we suggest that a decrease in *TBXAS1* expression causes the accumulation of AA, which subsequently activates the immune response through leukotrienes by leukocytes or monocytes, and it leads to increase the risk of depressive symptoms.

However, our findings must be scrutinized by further research because of the following limitations: First, genomic analyses were based on imputed data. rs6945590 was an imputed variant and not a genotyped variant; gene expression levels were predicted, and missing values of each item were interpolated. Although our findings should be demonstrated with complete data, such as whole genome sequencing and mRNA sequencing, we believe that they are not false positives because they were validated in independent datasets. Significant associations between rs6945590 or *TBXAS1* expression and the risk of depressive symptoms or depression were found in at least two independent cohorts. Second, the measures used to dichotomize participants differed between the cohorts used in the discovery and replication stages. Nevertheless, despite the possibility of different susceptible loci for these measures, rs6945590 consistently showed a significant association with depressive symptoms or depression across all cohorts. Third, the study did not have a sufficient sample size; therefore, we could not find the genomic loci associated with each heterogeneous symptom of depression and other variants that have relatively small effects on depressive symptoms in Koreans. Moreover, we may not have enough power to estimate genetic correlation between the symptoms. Further studies with larger sample sizes are needed to fully understand the genetic mechanism of depression and to determine whether *TBXAS1* could be used as a blood marker or target for treating depression.

In conclusion, a new causal genomic locus of depressive symptoms was identified in the Korean population. rs6945590 and *TBXAS1* were significantly associated with the risk of depressive symptoms. *TBXAS1* might play a role in the pathogenesis of depressive symptoms by affecting immune response. These findings might help to find novel markers or therapeutics for the diagnosis or treatment of depressive symptoms.

### Supplementary information

Supplementary Table

Supplementary Figures

## Data Availability

The data that support the findings of this study are available from the corresponding authors upon reasonable request.

## References

[1] Global Health Data Exchange (GHDx). http://ghdx.healthdata.org/gbd-results-tool?params=gbd-api-2019-permalink/d780dffbe8a381b25e1416884959e88b, Accessed 4 July 2022.

[2] Kim GE, Jo MW, Shin YW (2020). Increased prevalence of depression in South Korea from 2002 to 2013. Sci Rep..

[3] Chesney E, Goodwin GM, Fazel S (2014). Risks of all‐cause and suicide mortality in mental disorders: a meta‐review. World Psychiatry.

[4] OECD. *Health at a Glance 2021*, 2021.

[5] Howard DM, Adams MJ, Clarke T-K, Hafferty JD, Gibson J, Shirali M (2019). Genome-wide meta-analysis of depression identifies 102 independent variants and highlights the importance of the prefrontal brain regions. Nat Neurosci.

[6] Popejoy AB, Fullerton SM (2016). Genomics is failing on diversity. Nature.

[7] Peterson RE, Kuchenbaecker K, Walters RK, Chen C-Y, Popejoy AB, Periyasamy S (2019). Genome-wide association studies in ancestrally diverse populations: opportunities, methods, pitfalls, and recommendations. Cell.

[8] Cai N, Bigdeli TB, Kretzschmar W, Li Y, Liang J, Song L (2015). Sparse whole-genome sequencing identifies two loci for major depressive disorder. Nature.

[9] Giannakopoulou O, Lin K, Meng X, Su MH, Kuo PH, Peterson RE (2021). The genetic architecture of depression in individuals of East Asian ancestry: a genome-wide association study. JAMA Psychiatry.

[10] Hyde CL, Nagle MW, Tian C, Chen X, Paciga SA, Wendland JR (2016). Identification of 15 genetic loci associated with risk of major depression in individuals of European descent. Nat Genet.

[11] Howard DM, Adams MJ, Shirali M, Clarke TK, Marioni RE, Davies G (2018). Genome-wide association study of depression phenotypes in UK Biobank identifies variants in excitatory synaptic pathways. Nat Commun.

[12] Wray NR, Ripke S, Mattheisen M, Trzaskowski M, Byrne EM, Abdellaoui A (2018). Genome-wide association analyses identify 44 risk variants and refine the genetic architecture of major depression. Nat Genet.

[13] Thorp JG, Marees AT, Ong J-S, An J, MacGregor S, Derks EM (2020). Genetic heterogeneity in self-reported depressive symptoms identified through genetic analyses of the PHQ-9. Psychol Med.

[14] Lee C, Choe EK, Choi JM, Hwang Y, Lee Y, Park B (2018). Health and Prevention Enhancement (H-PEACE): a retrospective, population-based cohort study conducted at the Seoul National University Hospital Gangnam Center, Korea. BMJ Open.

[15] Moon S, Kim YJ, Han S, Hwang MY, Shin DM, Park MY (2019). The Korea Biobank Array: design and identification of coding variants associated with blood biochemical traits. Sci Rep..

[16] Seo S, Park K, Lee JJ, Choi KY, Lee KH, Won S (2019). SNP genotype calling and quality control for multi-batch-based studies. Genes Genomics.

[17] Beck AT, Ward CH, Mendelson M, Mock J, Erbaugh J (1961). An inventory for measuring depression. Arch Gen Psychiatry.

[18] Stekhoven DJ, Bühlmann P (2012). MissForest—non-parametric missing value imputation for mixed-type data. Bioinformatics.

[19] Beck AT, Steer RA, Carbin MG (1988). Psychometric properties of the Beck Depression Inventory: Twenty-five years of evaluation. Clin Psychol Rev.

[20] Anderson CA, Pettersson FH, Clarke GM, Cardon LR, Morris AP, Zondervan KT (2010). Data quality control in genetic case-control association studies. Nat Protoc.

[21] Das S, Forer L, Schönherr S, Sidore C, Locke AE, Kwong A (2016). Next-generation genotype imputation service and methods. Nat Genet.

[22] Purcell S, Neale B, Todd-Brown K, Thomas L, Ferreira MA, Bender D (2007). PLINK: a tool set for whole-genome association and population-based linkage analyses. Am J Hum Genet.

[23] Willer CJ, Li Y, Abecasis GR (2010). METAL: fast and efficient meta-analysis of genomewide association scans. Bioinformatics.

[24] Song YE, Lee S, Park K, Elston RC, Yang H-J, Won S (2018). ONETOOL for the analysis of family-based big data. Bioinformatics.

[25] Pruim RJ, Welch RP, Sanna S, Teslovich TM, Chines PS, Gliedt TP (2010). LocusZoom: regional visualization of genome-wide association scan results. Bioinformatics.

[26] Cho MJ, Kim KH (1998). Use of the center for epidemiologic studies depression (CES-D) scale in Korea. J Nerv Ment Dis.

[27] Weissman MM, Sholomskas D, Pottenger M, Prusoff BA, Locke BZ (1977). Assessing depressive symptoms in five psychiatric populations: a validation study. Am J Epidemiol.

[28] Lin S-H, Thakur R, Machiela MJ (2021). LDexpress: an online tool for integrating population-specific linkage disequilibrium patterns with tissue-specific expression data. BMC Bioinforma.

[29] Machiela MJ, Chanock SJ (2015). LDlink: a web-based application for exploring population-specific haplotype structure and linking correlated alleles of possible functional variants. Bioinformatics.

[30] Gamazon ER, Wheeler HE, Shah KP, Mozaffari SV, Aquino-Michaels K, Carroll RJ (2015). A gene-based association method for mapping traits using reference transcriptome data. Nat Genet.

[31] Zhu Z, Zheng Z, Zhang F, Wu Y, Trzaskowski M, Maier R (2018). Causal associations between risk factors and common diseases inferred from GWAS summary data. Nat Commun.

[32] Yang J, Lee SH, Goddard ME, Visscher PM (2011). GCTA: a tool for genome-wide complex trait analysis. Am J Hum Genet.

[33] Bowden J, Davey Smith G, Burgess S (2015). Mendelian randomization with invalid instruments: effect estimation and bias detection through Egger regression. Int J Epidemiol.

[34] Verbanck M, Chen CY, Neale B, Do R (2018). Detection of widespread horizontal pleiotropy in causal relationships inferred from Mendelian randomization between complex traits and diseases. Nat Genet.

[35] Yavorska OO, Burgess S (2017). MendelianRandomization: an R package for performing Mendelian randomization analyses using summarized data. Int J Epidemiol.

[36] Bowden J, Del Greco MF, Minelli C, Davey Smith G, Sheehan NA, Thompson JR (2016). Assessing the suitability of summary data for two-sample Mendelian randomization analyses using MR-Egger regression: the role of the I2 statistic. Int J Epidemiol.

[37] RexSoft Rex: Excel-based statistical analysis software. URL http://rexsoft.org/ (2018).

[38] Lee S, Yang J, Goddard M, Visscher P, Wray N (2012). Estimation of pleiotropy between complex diseases using SNP-derived genomic relationships and restricted maximum likelihood. Bioinformatics.

[39] Jung KS, Hong K-W, Jo HY, Choi J, Ban H-J, Cho SB (2020). KRGDB: the large-scale variant database of 1722 Koreans based on whole genome sequencing. Database.

[40] Karczewski KJ, Francioli LC, Tiao G, Cummings BB, Alföldi J, Wang Q (2020). The mutational constraint spectrum quantified from variation in 141,456 humans. Nature.

[41] Warden D, Rush AJ, Trivedi MH, Fava M, Wisniewski SR (2007). The STAR* D Project results: a comprehensive review of findings. Curr Psychiatry Rep..

[42] Miller AH, Maletic V, Raison CL (2009). Inflammation and its discontents: the role of cytokines in the pathophysiology of major depression. Biol Psychiatry.

[43] Arteaga-Henríquez G, Simon MS, Burger B, Weidinger E, Wijkhuijs A, Arolt V (2019). Low-grade inflammation as a predictor of antidepressant and anti-inflammatory therapy response in MDD patients: a systematic review of the literature in combination with an analysis of experimental data collected in the EU-MOODINFLAME consortium. Front Psychiatry.

[44] Branchi I, Poggini S, Capuron L, Benedetti F, Poletti S, Tamouza R (2021). Brain-immune crosstalk in the treatment of major depressive disorder. Eur Neuropsychopharmacol.

[45] Khandaker GM, Pearson RM, Zammit S, Lewis G, Jones PB (2014). Association of serum interleukin 6 and C-reactive protein in childhood with depression and psychosis in young adult life: a population-based longitudinal study. JAMA Psychiatry.

[46] Lamers F, Milaneschi Y, Smit JH, Schoevers RA, Wittenberg G, Penninx BW (2019). Longitudinal association between depression and inflammatory markers: results from the Netherlands study of depression and anxiety. Biol Psychiatry.

[47] Drevets WC, Wittenberg GM, Bullmore ET, Manji HK Immune targets for therapeutic development in depression: towards precision medicine. Nat Rev Drug Discov 2022;21:224–44. 10.1038/s41573-021-00368-1 PMC8763135 35039676

[48] Wang B, Wu L, Chen J, Dong L, Chen C, Wen Z (2021). Metabolism pathways of arachidonic acids: Mechanisms and potential therapeutic targets. Signal Transduct Target Ther.

[49] Lotrich FE, Sears B, McNamara RK (2013). Elevated ratio of arachidonic acid to long-chain omega-3 fatty acids predicts depression development following interferon-alpha treatment: relationship with interleukin-6. Brain Behav Immun.

[50] Adams PB, Lawson S, Sanigorski A, Sinclair AJ (1996). Arachidonic acid to eicosapentaenoic acid ratio in blood correlates positively with clinical symptoms of depression. Lipids.

[51] Kessing LV, Rytgaard HC, Gerds T, Berk M, Ekstrøm C, Andersen P (2019). New drug candidates for depression–a nationwide population‐based study. Acta Psychiatr Scand.

[52] Berk M, Mohebbi M, Dean OM, Cotton SM, Chanen AM, Dodd S (2020). Youth Depression Alleviation with Anti-inflammatory Agents (YoDA-A): a randomised clinical trial of rosuvastatin and aspirin. BMC Med.

[53] Berk M, Agustini B, Woods RL, Nelson MR, Shah RC, Reid CM (2021). Effects of aspirin on the long-term management of depression in older people: a double-blind randomised placebo-controlled trial. Mol Psychiatry.

[54] Peters-Golden M, Canetti C, Mancuso P, Coffey MJ (2005). Leukotrienes: underappreciated mediators of innate immune responses. J Immunol.

[55] Yu XB, Dong RR, Wang H, Lin JR, An YQ, Du Y (2016). Knockdown of hippocampal cysteinyl leukotriene receptor 1 prevents depressive behavior and neuroinflammation induced by chronic mild stress in mice. Psychopharmacology.

[56] Cereza G, Doladé NG, Laporte JR (2012). Nightmares induced by montelukast in children and adults. Eur Respiratory J.

[57] Hara H, Sugahara K, Hashimoto M, Mikuriya T, Tahara S, Yamashita H (2014). Effectiveness of the leukotriene receptor antagonist pranlukast hydrate for the treatment of sleep disorder in patients with perennial allergic rhinitis. Acta Oto-Laryngologica.

[58] Haeggström JZ (2018). Leukotriene biosynthetic enzymes as therapeutic targets. J Clin Investig.

[59] Seidel A, Arolt V, Hunstiger M, Rink L, Behnisch A, Kirchner H (1996). Major depressive disorder is associated with elevated monocyte counts. Acta Psychiatr Scand.

[60] Sealock JM, Lee YH, Moscati A, Venkatesh S, Voloudakis G, Straub P (2021). Use of the PsycheMERGE network to investigate the association between depression polygenic scores and white blood cell count. JAMA Psychiatry.

