# Supplementary material for: Genome-wide association study implicates the role of TBXAS1 in the pathogenesis of depressive symptoms among the Korean population
Source: Transl Psychiatry. 2024 Feb 6;14:80. doi: 10.1038/s41398-024-02777-3 (PMC10847124; doi:10.1038/s41398-024-02777-3)
Supplement: Supplementary file 1 — Supplementary Table [file 41398_2024_2777_MOESM1_ESM.docx]

**Supplementary Tables**

**Table S1**. GWAS results of H-PEACE cohort. Chr, chromosome; BP, base pair; OR, odds ratio; CI, confidence interval.

| Chr | Region^1^  (Mb) | Variant^2^ | Location  (BP) | Effect / Other  Alleles | OR  (95% CI) | *P*-value | INFO^3^ |
| --- | --- | --- | --- | --- | --- | --- | --- |
| 12 | 122.3-122.5 | rs745327 | 122456524 | C / T | 0.80  (0.74-0.88) | $7.61\times{10}^{-7}$ | 0.828 |
| 22 | 26.3-26.3 | rs2331164 | 26293153 | A / G | 0.80  (0.73-0.88) | $1.30\times{10}^{-6}$ | 0.995 |
| 18 | - | rs62102340 | 70960895 | C / T | 0.80  (0.73-0.88) | $1.53\times{10}^{-6}$ | 0.982 |
| 5 | 158.4-158.4 | rs78864733 | 158401752 | G / A | 1.45  (1.23-1.70) | $2.02\times{10}^{-6}$ | 0.978 |
| 14 | 32.5-32.5 | rs55732934 | 32519789 | C / A | 0.80  (0.73-0.88) | $2.24\times{10}^{-6}$ | 0.847 |
| 6 | 66.3-66.3 | rs354400 | 66315591 | G / T | 1.23  (1.13-1.33) | $2.53\times{10}^{-6}$ | 0.929 |
| 14 | - | rs7155737 | 89898507 | G / A | 0.80  (0.72-0.88) | $3.39\times{10}^{-6}$ | 0.968 |
| 6 | 81.7-81.7 | rs73750927 | 81659187 | A / G | 1.33  (1.18-1.49) | $3.55\times{10}^{-6}$ | 0.948 |
| 7 | 139.7-139.7 | rs6945590 | 139762282 | G / A | 0.82  (0.75-0.89) | $3.79\times{10}^{-6}$ | 0.833 |
| 1 | 58.2-58.2 | rs10493232 | 58229635 | G / C | 0.82  (0.75-0.89) | $4.15\times{10}^{-6}$ | 0.852 |
| 12 | - | rs10083197 | 13285683 | G / C | 0.81  (0.74-0.89) | $4.62\times{10}^{-6}$ | 0.889 |
| 3 | - | rs1502172 | 135235358 | A / G | 0.80  (0.73-0.88) | $4.70\times{10}^{-6}$ | 0.982 |
| 5 | - | rs78880954 | 106615631 | C / A | 1.51  (1.26-1.80) | $4.82\times{10}^{-6}$ | 0.983 |
| 10 | - | rs10824840 | 54697447 | G / T | 1.22  (1.12-1.32) | $6.36\times{10}^{-6}$ | 0.845 |
| 16 | - | rs55639911 | 87677591 | C / A | 1.24  (1.13-1.37) | $9.16\times{10}^{-6}$ | 0.853 |
| 8 | 143.1-143.1 | rs4917294 | 143119767 | A / C | 1.21  (1.11-1.32) | $9.52\times{10}^{-6}$ | 0.945 |

^1^Loci where putatively significant variants (*P* < 1 × 10^-5^) locates based on GRCh37. Dashed lines represent where only one variant exists.

^2^Most significant variant in the region.

^3^Imputation quality score.

**Table S2**. GWAS results of GENIE cohort. Chr, chromosome; BP, base pair; OR, odds ratio; CI, confidence interval.

| Chr | Region^1^  (Mb) | Variant^2^ | Location  (BP) | Effect / Other  Alleles | OR  (95% CI) | *P*-value | INFO^3^ |
| --- | --- | --- | --- | --- | --- | --- | --- |
| 1 | - | rs16826069 | 39797055 | G / A | 0.66  (0.55-0.78) | $2.13\times{10}^{-6}$ | 0.999 |
| 9 | 114.7-114.7 | rs10123358 | 114731169 | C / G | 1.54  (1.28-1.85) | $4.80\times{10}^{-6}$ | 0.944 |
| 5 | 138.3-138.3 | rs12108892 | 138299630 | A / G | 1.58  (1.30-1.92) | $5.31\times{10}^{-6}$ | 0.903 |
| 5 | - | rs62374668 | 124680323 | C / G | 1.57  (1.29-1.91) | $5.50\times{10}^{-6}$ | 0.870 |
| 10 | 2.36-2.36 | rs4567377 | 2355223 | T / A | 0.69  (0.59-0.81) | $6.18\times{10}^{-6}$ | 0.831 |
| 1 | - | rs73063174 | 199786268 | T / C | 1.99  (1.47-2.69) | $6.84\times{10}^{-6}$ | 0.960 |
| 16 | - | rs35557824 | 84466728 | A / G | 1.69  (1.34-2.14) | $9.72\times{10}^{-6}$ | 0.943 |

^1^Loci where putatively significant variants (*P* < 1 × 10^-5^) locates based on GRCh37. Dashed lines represent where only one variant exists.

^2^Most significant variant in the region.

^3^Imputation quality score.

**Table S3.** Traits significantly associated with variants in LD region of rs6945590 in East Asian population (*P* < 5.00 × 10^−8^; R^2^ > 0.8) by LDtrait.

| GWAS trait | Variant | Position  (GRCh37) | $R^{2}$ | *P*-value |
| --- | --- | --- | --- | --- |
| Height | rs7781964 | chr7:139749346 | 0.833 | 3.00E-24 |
| White blood cell count | rs2269996 | chr7:139724358 | 0.822 | 1.00E-14 |
| Monocyte count | rs2269996 | chr7:139724358 | 0.822 | 7.00E-14 |
| Lymphocyte counts | rs7781964 | chr7:139749346 | 0.833 | 6.00E-11 |
| Coronary artery disease | rs10237377 | chr7:139757136 | 0.826 | 9.00E-11 |
| Myocardial infarction | rs9640375 | chr7:139737344 | 0.818 | 2.00E-10 |
| Coronary artery disease | rs10237377 | chr7:139757136 | 0.826 | 7.00E-09 |
| Coronary artery disease | rs2269997 | chr7:139723400 | 0.815 | 1.00E-08 |

**Table S4.** Genes for which rs6945590 is an eQTL by LDexpress. chr, chromosome.

| Query | Variant | Position  (GRCh37) | $R^{2}$ | $D^{'}$ | Gene  Symbol | Tissue | Effect / Other  Alleles | Effect  Size | *P*-value |
| --- | --- | --- | --- | --- | --- | --- | --- | --- | --- |
| rs6945590 | rs6945590 | chr7:139762282 | 1.0 | 1.0 | PARP12 | Nerve - Tibial | G / A | -0.196 | $2\times{10}^{-6}$ |
| rs6945590 | rs6945590 | chr7:139762282 | 1.0 | 1.0 | PARP12 | Testis | G / A | 0.195 | $6\times{10}^{-6}$ |

**Table S5.** Relationship between rs10242990 and *TBXAS1* expression by LDexpress.

| Variant | Position  (GRCh37) | $R^{2}$^1^ | Gene  Symbol | Tissue | Effect / Other  Alleles | Haplotypes^2^  (%) | Effect  Size | *P*-value |
| --- | --- | --- | --- | --- | --- | --- | --- | --- |
| rs10242990 | chr7:139730500 | 0.807 | *TBXAS1* | Whole Blood | G / C | A_G (51%)  G_C (44%)  A_C (3%)  G_G (2%) | -0.098 | $9\times{10}^{-15}$ |

^1^Correlation with rs6945990 and the variant

^2^Incidence ratio of allele pairs between alleles of rs6945590 (the former) and rs10242990 (the latter)

**Table S6.** Association between variants in LD region of rs6945590 and expression level of genes by LDexpress. chr, chromosome.

| Variant | Position  (GRCh37) | $R^{2}$ | Gene  Symbol | Tissue | Effect / Other  Alleles | Effect  Size | *P*-value |
| --- | --- | --- | --- | --- | --- | --- | --- |
| rs10242990 | chr7:139730500 | 0.807 | TBXAS1 | Whole Blood | G / C | -0.098 | 8.85E-15 |
| rs10156003 | chr7:139756458 | 0.822 | TBXAS1 | Whole Blood | T / A | -0.089 | 9.83E-13 |
| rs10274187 | chr7:139748739 | 0.822 | TBXAS1 | Whole Blood | G / C | -0.088 | 1.41E-12 |
| rs10952541 | chr7:139749058 | 0.818 | TBXAS1 | Whole Blood | T / G | -0.088 | 1.41E-12 |
| rs12666816 | chr7:139751352 | 0.822 | TBXAS1 | Whole Blood | G / A | -0.088 | 1.41E-12 |
| rs10241819 | chr7:139753080 | 0.822 | TBXAS1 | Whole Blood | C / T | -0.088 | 1.41E-12 |
| rs2362460 | chr7:139754451 | 0.822 | TBXAS1 | Whole Blood | C / T | -0.088 | 1.41E-12 |
| rs7810536 | chr7:139754819 | 0.822 | TBXAS1 | Whole Blood | C / T | -0.088 | 1.41E-12 |
| rs10253126 | chr7:139758053 | 0.822 | TBXAS1 | Whole Blood | T / C | -0.088 | 1.41E-12 |
| rs13760 | chr7:139724169 | 0.811 | TBXAS1 | Whole Blood | G / A | -0.088 | 1.47E-12 |
| rs2362462 | chr7:139760010 | 0.822 | TBXAS1 | Whole Blood | C / T | -0.088 | 1.54E-12 |
| rs2040969 | chr7:139727670 | 0.804 | TBXAS1 | Whole Blood | C / T | -0.088 | 2.62E-12 |
| rs10237377 | chr7:139757136 | 0.826 | TBXAS1 | Whole Blood | T / G | -0.088 | 3.18E-12 |
| rs6945359 | chr7:139758476 | 0.826 | TBXAS1 | Whole Blood | T / C | -0.088 | 3.18E-12 |
| rs10155912 | chr7:139758721 | 0.826 | TBXAS1 | Whole Blood | C / A | -0.088 | 3.18E-12 |
| rs757833 | chr7:139734697 | 0.818 | TBXAS1 | Whole Blood | A / C | -0.086 | 1.13E-11 |
| rs7794528 | chr7:139761463 | 0.83 | TBXAS1 | Whole Blood | T / C | -0.075 | 4.80E-09 |
| rs2269997 | chr7:139723400 | 0.815 | TBXAS1 | Whole Blood | A / G | -0.072 | 1.44E-06 |
| rs2286195 | chr7:139728584 | 0.804 | TBXAS1 | Whole Blood | C / A | -0.071 | 1.79E-06 |
| rs9640375 | chr7:139737344 | 0.818 | TBXAS1 | Whole Blood | T / C | -0.07 | 2.21E-06 |
| rs35586793 | chr7:139722530 | 0.815 | TBXAS1 | Whole Blood | A / T | -0.069 | 3.39E-06 |
| rs142432643 | chr7:139760540 | 0.826 | TBXAS1 | Whole Blood | T / TCCCTGCTCTC | -0.069 | 4.07E-06 |
| rs10435198 | chr7:139758615 | 0.826 | TBXAS1 | Whole Blood | C / G | -0.067 | 6.00E-06 |
| rs2001621 | chr7:139760002 | 0.825 | TBXAS1 | Whole Blood | T / C | -0.067 | 6.00E-06 |
| rs10435199 | chr7:139758826 | 0.826 | TBXAS1 | Whole Blood | T / C | -0.066 | 7.86E-06 |
| rs2362462 | chr7:139760010 | 0.822 | PARP12 | Testis | C / T | -0.271 | 2.72E-14 |
| rs10274187 | chr7:139748739 | 0.822 | PARP12 | Testis | G / C | -0.262 | 2.04E-13 |
| rs10952541 | chr7:139749058 | 0.818 | PARP12 | Testis | T / G | -0.262 | 2.04E-13 |
| rs12666816 | chr7:139751352 | 0.822 | PARP12 | Testis | G / A | -0.262 | 2.04E-13 |
| rs10241819 | chr7:139753080 | 0.822 | PARP12 | Testis | C / T | -0.262 | 2.04E-13 |
| rs2362460 | chr7:139754451 | 0.822 | PARP12 | Testis | C / T | -0.262 | 2.04E-13 |
| rs7810536 | chr7:139754819 | 0.822 | PARP12 | Testis | C / T | -0.262 | 2.04E-13 |
| rs10156003 | chr7:139756458 | 0.822 | PARP12 | Testis | T / A | -0.262 | 2.04E-13 |
| rs10253126 | chr7:139758053 | 0.822 | PARP12 | Testis | T / C | -0.262 | 2.04E-13 |
| rs10237377 | chr7:139757136 | 0.826 | PARP12 | Testis | T / G | -0.253 | 3.23E-13 |
| rs6945359 | chr7:139758476 | 0.826 | PARP12 | Testis | T / C | -0.253 | 3.23E-13 |
| rs10155912 | chr7:139758721 | 0.826 | PARP12 | Testis | C / A | -0.253 | 3.23E-13 |
| rs13760 | chr7:139724169 | 0.811 | PARP12 | Testis | G / A | -0.259 | 5.93E-13 |
| rs757833 | chr7:139734697 | 0.818 | PARP12 | Testis | A / C | -0.251 | 7.37E-13 |
| rs2040969 | chr7:139727670 | 0.804 | PARP12 | Testis | C / T | -0.251 | 8.07E-13 |
| rs7794528 | chr7:139761463 | 0.83 | PARP12 | Testis | T / C | -0.242 | 7.04E-12 |
| rs10242990 | chr7:139730500 | 0.807 | PARP12 | Testis | G / C | -0.203 | 1.69E-08 |
| rs737321 | chr7:139762273 | 0.984 | PARP12 | Testis | G / A | 0.205 | 4.17E-06 |
| rs2362462 | chr7:139760010 | 0.822 | PARP12 | Skin - Sun Exposed (Lower leg) | C / T | 0.226 | 1.46E-11 |
| rs10156003 | chr7:139756458 | 0.822 | PARP12 | Skin - Sun Exposed (Lower leg) | T / A | 0.226 | 1.54E-11 |
| rs13760 | chr7:139724169 | 0.811 | PARP12 | Skin - Sun Exposed (Lower leg) | G / A | 0.224 | 3.14E-11 |
| rs10274187 | chr7:139748739 | 0.822 | PARP12 | Skin - Sun Exposed (Lower leg) | G / C | 0.222 | 3.32E-11 |
| rs10952541 | chr7:139749058 | 0.818 | PARP12 | Skin - Sun Exposed (Lower leg) | T / G | 0.222 | 3.32E-11 |
| rs12666816 | chr7:139751352 | 0.822 | PARP12 | Skin - Sun Exposed (Lower leg) | G / A | 0.222 | 3.32E-11 |
| rs10241819 | chr7:139753080 | 0.822 | PARP12 | Skin - Sun Exposed (Lower leg) | C / T | 0.222 | 3.32E-11 |
| rs2362460 | chr7:139754451 | 0.822 | PARP12 | Skin - Sun Exposed (Lower leg) | C / T | 0.222 | 3.32E-11 |
| rs7810536 | chr7:139754819 | 0.822 | PARP12 | Skin - Sun Exposed (Lower leg) | C / T | 0.222 | 3.32E-11 |
| rs10253126 | chr7:139758053 | 0.822 | PARP12 | Skin - Sun Exposed (Lower leg) | T / C | 0.222 | 3.32E-11 |
| rs10237377 | chr7:139757136 | 0.826 | PARP12 | Skin - Sun Exposed (Lower leg) | T / G | 0.179 | 1.64E-07 |
| rs6945359 | chr7:139758476 | 0.826 | PARP12 | Skin - Sun Exposed (Lower leg) | T / C | 0.179 | 1.64E-07 |
| rs10155912 | chr7:139758721 | 0.826 | PARP12 | Skin - Sun Exposed (Lower leg) | C / A | 0.179 | 1.64E-07 |
| rs757833 | chr7:139734697 | 0.818 | PARP12 | Skin - Sun Exposed (Lower leg) | A / C | 0.175 | 4.19E-07 |
| rs2040969 | chr7:139727670 | 0.804 | PARP12 | Skin - Sun Exposed (Lower leg) | C / T | 0.175 | 4.48E-07 |
| rs10242990 | chr7:139730500 | 0.807 | PARP12 | Skin - Sun Exposed (Lower leg) | G / C | 0.155 | 8.44E-06 |
| rs10274187 | chr7:139748739 | 0.822 | PARP12 | Skin - Not Sun Exposed (Suprapubic) | G / C | 0.285 | 1.41E-13 |
| rs10952541 | chr7:139749058 | 0.818 | PARP12 | Skin - Not Sun Exposed (Suprapubic) | T / G | 0.285 | 1.41E-13 |
| rs12666816 | chr7:139751352 | 0.822 | PARP12 | Skin - Not Sun Exposed (Suprapubic) | G / A | 0.285 | 1.41E-13 |
| rs10241819 | chr7:139753080 | 0.822 | PARP12 | Skin - Not Sun Exposed (Suprapubic) | C / T | 0.285 | 1.41E-13 |
| rs2362460 | chr7:139754451 | 0.822 | PARP12 | Skin - Not Sun Exposed (Suprapubic) | C / T | 0.285 | 1.41E-13 |
| rs7810536 | chr7:139754819 | 0.822 | PARP12 | Skin - Not Sun Exposed (Suprapubic) | C / T | 0.285 | 1.41E-13 |
| rs10156003 | chr7:139756458 | 0.822 | PARP12 | Skin - Not Sun Exposed (Suprapubic) | T / A | 0.285 | 1.41E-13 |
| rs10253126 | chr7:139758053 | 0.822 | PARP12 | Skin - Not Sun Exposed (Suprapubic) | T / C | 0.285 | 1.41E-13 |
| rs2362462 | chr7:139760010 | 0.822 | PARP12 | Skin - Not Sun Exposed (Suprapubic) | C / T | 0.285 | 1.41E-13 |
| rs13760 | chr7:139724169 | 0.811 | PARP12 | Skin - Not Sun Exposed (Suprapubic) | G / A | 0.282 | 3.28E-13 |
| rs10237377 | chr7:139757136 | 0.826 | PARP12 | Skin - Not Sun Exposed (Suprapubic) | T / G | 0.248 | 3.11E-10 |
| rs6945359 | chr7:139758476 | 0.826 | PARP12 | Skin - Not Sun Exposed (Suprapubic) | T / C | 0.248 | 3.11E-10 |
| rs10155912 | chr7:139758721 | 0.826 | PARP12 | Skin - Not Sun Exposed (Suprapubic) | C / A | 0.248 | 3.11E-10 |
| rs757833 | chr7:139734697 | 0.818 | PARP12 | Skin - Not Sun Exposed (Suprapubic) | A / C | 0.245 | 8.04E-10 |
| rs2040969 | chr7:139727670 | 0.804 | PARP12 | Skin - Not Sun Exposed (Suprapubic) | C / T | 0.242 | 1.19E-09 |
| rs10242990 | chr7:139730500 | 0.807 | PARP12 | Skin - Not Sun Exposed (Suprapubic) | G / C | 0.235 | 3.61E-09 |
| rs7794528 | chr7:139761463 | 0.83 | PARP12 | Skin - Not Sun Exposed (Suprapubic) | T / C | 0.216 | 1.76E-07 |
| rs35586793 | chr7:139722530 | 0.815 | PARP12 | Prostate | A / T | 0.277 | 3.41E-06 |
| rs2269997 | chr7:139723400 | 0.815 | PARP12 | Prostate | A / G | 0.277 | 3.41E-06 |
| rs9640375 | chr7:139737344 | 0.818 | PARP12 | Prostate | T / C | 0.277 | 3.41E-06 |
| rs7805521 | chr7:139761176 | 0.829 | PARP12 | Prostate | A / T | 0.278 | 7.85E-06 |
| rs7801310 | chr7:139761248 | 0.837 | PARP12 | Prostate | G / A | 0.279 | 8.59E-06 |
| rs2269996 | chr7:139724358 | 0.822 | PARP12 | Prostate | A / G | 0.278 | 9.33E-06 |
| rs7801310 | chr7:139761248 | 0.837 | PARP12 | Nerve - Tibial | G / A | 0.238 | 3.54E-08 |
| rs7805521 | chr7:139761176 | 0.829 | PARP12 | Nerve - Tibial | A / T | 0.23 | 8.13E-08 |
| rs9640375 | chr7:139737344 | 0.818 | PARP12 | Nerve - Tibial | T / C | 0.217 | 2.11E-07 |
| rs35586793 | chr7:139722530 | 0.815 | PARP12 | Nerve - Tibial | A / T | 0.213 | 2.86E-07 |
| rs7781964 | chr7:139749346 | 0.833 | PARP12 | Nerve - Tibial | A / G | 0.217 | 3.22E-07 |
| rs2269997 | chr7:139723400 | 0.815 | PARP12 | Nerve - Tibial | A / G | 0.214 | 3.24E-07 |
| rs2286196 | chr7:139727135 | 0.822 | PARP12 | Nerve - Tibial | A / G | 0.217 | 3.69E-07 |
| rs2269996 | chr7:139724358 | 0.822 | PARP12 | Nerve - Tibial | A / G | 0.218 | 3.83E-07 |
| rs10435199 | chr7:139758826 | 0.826 | PARP12 | Nerve - Tibial | T / C | 0.209 | 5.26E-07 |
| rs10435198 | chr7:139758615 | 0.826 | PARP12 | Nerve - Tibial | C / G | 0.208 | 7.56E-07 |
| rs2001621 | chr7:139760002 | 0.825 | PARP12 | Nerve - Tibial | T / C | 0.208 | 7.56E-07 |
| rs2286195 | chr7:139728584 | 0.804 | PARP12 | Nerve - Tibial | C / A | 0.207 | 8.78E-07 |
| rs142432643 | chr7:139760540 | 0.826 | PARP12 | Nerve - Tibial | T / TCCCTGCTCTC | 0.203 | 1.38E-06 |
| rs6969159 | chr7:139745791 | 0.83 | PARP12 | Nerve - Tibial | A / G | 0.208 | 1.55E-06 |
| rs10242990 | chr7:139730500 | 0.807 | PARP12 | Nerve - Tibial | G / C | 0.171 | 3.18E-06 |
| rs737321 | chr7:139762273 | 0.984 | PARP12 | Nerve - Tibial | G / A | -0.193 | 5.25E-06 |
| rs2362462 | chr7:139760010 | 0.822 | PARP12 | Esophagus - Mucosa | C / T | 0.152 | 7.27E-08 |
| rs10274187 | chr7:139748739 | 0.822 | PARP12 | Esophagus - Mucosa | G / C | 0.151 | 8.25E-08 |
| rs10952541 | chr7:139749058 | 0.818 | PARP12 | Esophagus - Mucosa | T / G | 0.151 | 8.25E-08 |
| rs12666816 | chr7:139751352 | 0.822 | PARP12 | Esophagus - Mucosa | G / A | 0.151 | 8.25E-08 |
| rs10241819 | chr7:139753080 | 0.822 | PARP12 | Esophagus - Mucosa | C / T | 0.151 | 8.25E-08 |
| rs2362460 | chr7:139754451 | 0.822 | PARP12 | Esophagus - Mucosa | C / T | 0.151 | 8.25E-08 |
| rs7810536 | chr7:139754819 | 0.822 | PARP12 | Esophagus - Mucosa | C / T | 0.151 | 8.25E-08 |
| rs10156003 | chr7:139756458 | 0.822 | PARP12 | Esophagus - Mucosa | T / A | 0.151 | 8.25E-08 |
| rs10253126 | chr7:139758053 | 0.822 | PARP12 | Esophagus - Mucosa | T / C | 0.151 | 8.25E-08 |
| rs13760 | chr7:139724169 | 0.811 | PARP12 | Esophagus - Mucosa | G / A | 0.143 | 4.43E-07 |

**Table S7.** Genes whose predicted expression level was significantly associated with risk of depressive symptoms in H-PEACE cohort. Chr, chromosome.

| Gene Symbol | Ensemble Gene ID | Chr | H-PEACE | | | GENIE | |
| --- | --- | --- | --- | --- | --- | --- | --- |
|  |  |  | Estimate | *P*-value | FDR | Estimate | *P*-value |
| - | ENSG00000235878 | 21 | 0.034 | 1.87 × 10^−5^ | 1.65 × 10^−3^ | 0.007 | 0.297 |
| *TBXAS1* | ENSG00000059377 | 7 | -0.023 | 3.54 × 10^−5^ | 1.26 × 10^−2^ | -0.023 | 2.11 × 10^−2^ |
| *HPD* | ENSG00000158104 | 12 | 0.036 | 4.38 × 10^−5^ | 1.52 × 10^−2^ | -0.018 | 0.243 |
| *LSS* | ENSG00000160285 | 21 | 0.046 | 1.43 × 10^−4^ | 6.30 × 10^−3^ | 0.033 | 8.55 × 10^−2^ |
| *CYYR1* | ENSG00000166265 | 21 | 0.009 | 7.98 × 10^−4^ | 2.34 × 10^−2^ | 0.003 | 0.525 |
| *YBEY* | ENSG00000182362 | 21 | -0.031 | 3.40 × 10^−3^ | 7.48 × 10^−2^ | -0.038 | 4.92 × 10^−2^ |
| *SPATC1L* | ENSG00000160284 | 21 | -0.016 | 5.50 × 10^−3^ | 9.69 × 10^−2^ | -0.004 | 0.691 |

**Table S8.** The results of Mendelian randomization analyses (other than GSMR) examining the causal relationship between *TBXAS1* expression and the risk of depressive symptoms. SE, standard error; CI, confidence interval.

| Method | Estimates | SE | $95\%$ CI | *P*-value |
| --- | --- | --- | --- | --- |
| MR-PRESSO | -0.835 | 0.199 | -1.224, -0.446 | $1.22\times{10}^{-3}$ |
| Simple median | -0.962 | 0.698 | -2.329, 0.405 | 0.168 |
| Weighted median | -0.679 | 0.276 | -1.220, -0.139 | $1.38\times{10}^{-2}$ |
| Penalized weighted median | -0.679 | 0.276 | -1.220, -0.139 | $1.38\times{10}^{-2}$ |
| IVW | -0.835 | 0.250 | -1.326, -0.344 | $8.55\times{10}^{-4}$ |
| Penalized IVW | -0.835 | 0.250 | -1.326, -0.344 | $8.55\times{10}^{-4}$ |
| Robust IVW | -0.766 | 0.219 | -1.195, -0.338 | $4.57\times{10}^{-4}$ |
| Penalized robust IVW | -0.766 | 0.219 | -1.195, -0.338 | $4.57\times{10}^{-4}$ |
| MR-Egger | -0.523 | 0.359 | -1.226, 0.180 | 0.145 |
| Penalized MR-Egger | -0.523 | 0.359 | -1.226, 0.180 | 0.145 |
| Robust MR-Egger | -0.537 | 0.152 | -0.835, -0.238 | $4.33\times{10}^{-4}$ |
| Penalized Robust MR-Egger | -0.537 | 0.152 | -0.835, -0.238 | $4.33\times{10}^{-4}$ |

**Table S9.** Results of association analysis between rs6945590 and risk of depressive symptoms in each cluster. OR, odds ratio; CI, confidence interval.

| Cluster | Item | *P*-value | OR  (95% CI) |
| --- | --- | --- | --- |
| 1 | 19 | 0.2734 | 1.05  (0.96-1.14) |
| 2 | 6 | 0.3014 | 1.07  (0.94-1.22) |
| 3 | 1, 2, 3, 9, 10 | 5.32 × 10^−2^ | 1.08  (1.00-1.17) |
| 4 | 4, 5, 7, 8, 11, 12, 13, 14, 15, 16, 17, 18, 20, 21 | 1.95 × 10^−8^ | 1.22  (1.14-1.31) |

**Table S10.** Compare results of significant variants in European study with those of meta-analysis of H-PEACE and GENIE cohorts. OR, odds ratio.

| Variant | Position  (GRCh37) | A1 | A2 | OR  (Howard et al^1^) | OR  (meta-analysis of  H-PEACE and GENIE) | *P*-value  (meta-analysis of  H-PEACE and GENIE) |
| --- | --- | --- | --- | --- | --- | --- |
| rs301799 | chr1:8489302 | T | C | 0.975 | 1.026 | 0.6076 |
| rs1002656 | chr1:37192741 | T | C | 0.979 | 1.001 | 0.9869 |
| rs11579246 | chr1:50559162 | A | G | 1.033 | 1.014 | 0.7134 |
| rs2568958 | chr1:72765116 | A | G | 1.034 | 0.916 | 0.2385 |
| rs10890020 | chr1:73668836 | A | G | 0.974 | 1.012 | 0.7867 |
| rs113188507 | chr1:80809636 | A | G | 1.024 | 1.219 | 0.01109 |
| rs10913112 | chr1:175913828 | T | C | 0.979 | 1.028 | 0.4697 |
| rs72710803 | chr1:177428018 | A | C | 0.976 | 0.98 | 0.7924 |
| rs17641524 | chr1:197704717 | T | C | 0.976 | 1.025 | 0.7234 |
| rs12052908 | chr2:22503044 | A | T | 0.979 | 1.063 | 0.1303 |
| rs1568452 | chr2:58012833 | T | C | 1.03 | 1.027 | 0.4745 |
| rs1226412 | chr2:157111313 | T | C | 1.023 | 1.011 | 0.7957 |
| rs62188629 | chr2:208044470 | A | G | 1.017 | 0.969 | 0.593 |
| rs1095626 | chr3:157977962 | T | C | 0.972 | 1.062 | 0.1191 |
| rs7685686 | chr4:3207142 | A | G | 1.017 | 0.99 | 0.7782 |
| rs35553410 | chr4:131237381 | T | C | 0.984 | 1.066 | 0.1664 |
| rs7659414 | chr4:177350956 | A | C | 0.991 | 0.988 | 0.7677 |
| rs60157091 | chr5:61509655 | T | C | 1.021 | 1.069 | 0.08227 |
| rs10061069 | chr5:93071630 | C | G | 0.975 | 1.056 | 0.2461 |
| rs11135349 | chr5:164523472 | A | C | 0.975 | 0.968 | 0.5074 |
| rs200949 | chr6:27835435 | A | G | 1.044 | 0.981 | 0.7744 |
| rs7758630 | chr6:101387304 | A | T | 0.984 | 1.053 | 0.1788 |
| rs2876520 | chr6:142996618 | C | G | 0.981 | 0.998 | 0.9603 |
| rs725616 | chr6:147950422 | T | C | 1.014 | 0.947 | 0.1405 |
| rs2029865 | chr6:165121844 | A | T | 0.982 | 0.943 | 0.2056 |
| rs3823624 | chr7:2110346 | T | C | 1.03 | 1.036 | 0.5299 |
| rs2043539 | chr7:12253880 | A | G | 1.022 | 0.996 | 0.9129 |
| rs16887442 | chr7:82936909 | T | C | 1.015 | 0.971 | 0.4259 |
| rs7807677 | chr7:117502574 | T | C | 1.021 | 0.971 | 0.4257 |
| rs7837935 | chr8:65562019 | T | G | 0.978 | 0.947 | 0.1734 |
| rs263645 | chr9:17016503 | A | T | 1.018 | 1.027 | 0.4695 |
| rs3793577 | chr9:23737627 | A | G | 0.979 | 1.013 | 0.725 |
| rs7030813 | chr9:36999369 | T | C | 1.025 | 1.029 | 0.4558 |
| rs10817969 | chr9:119731045 | T | G | 1.017 | 0.907 | 0.01348 |
| rs2670139 | chr9:126634255 | T | C | 0.982 | 1.075 | 0.1393 |
| rs997934 | chr10:1795194 | T | C | 1.016 | 0.964 | 0.333 |
| rs1448938 | chr11:30892824 | A | G | 1.017 | 1.028 | 0.4917 |
| rs2509805 | chr11:57650796 | T | C | 1.019 | 0.981 | 0.7333 |
| rs58621819 | chr11:65314830 | A | T | 0.983 | 0.989 | 0.8354 |
| rs7117514 | chr11:70544937 | A | G | 0.984 | 1.035 | 0.4094 |
| rs57344483 | chr11:127022560 | A | G | 0.976 | 0.98 | 0.6279 |
| rs56314503 | chr12:84465022 | T | G | 0.98 | 1.115 | 0.02056 |
| rs3213572 | chr12:121205078 | A | G | 1.02 | 1.02 | 0.601 |
| rs9592461 | chr13:66941792 | A | G | 1.025 | 1.114 | 0.007403 |
| rs4772087 | chr13:99115041 | T | C | 1.021 | 1.034 | 0.3755 |
| rs1956373 | chr14:60141822 | T | G | 0.983 | 1.04 | 0.3222 |
| rs1152578 | chr14:64697037 | T | C | 0.985 | 0.963 | 0.3409 |
| rs1045430 | chr14:75130235 | T | G | 0.978 | 0.955 | 0.2364 |
| rs10149470 | chr14:104017953 | A | G | 0.98 | 0.97 | 0.4273 |
| rs8037355 | chr15:37643831 | T | C | 0.981 | 0.947 | 0.1674 |
| rs7198928 | chr16:7666402 | T | C | 1.022 | 1.034 | 0.4436 |
| rs7200826 | chr16:13066833 | T | C | 1.027 | 0.941 | 0.4376 |
| rs56887639 | chr16:13755530 | A | G | 0.983 | 0.98 | 0.7051 |
| rs12923444 | chr16:21639710 | A | C | 0.977 | 1.04 | 0.5452 |
| rs12967855 | chr18:35138245 | A | G | 1.021 | 0.967 | 0.3869 |
| rs12967143 | chr18:53099012 | C | G | 0.975 | 0.953 | 0.197 |
| rs7241572 | chr18:77580712 | A | G | 1.02 | 0.99 | 0.8653 |
| rs33431 | chr19:30939989 | T | C | 1.012 | 0.993 | 0.8775 |
| rs12624433 | chr20:44680853 | A | G | 1.019 | 0.995 | 0.9065 |

**References**

1. Howard DM, Adams MJ, Clarke T-K, Hafferty JD, Gibson J, Shirali M *et al.* Genome-wide meta-analysis of depression identifies 102 independent variants and highlights the importance of the prefrontal brain regions. *Nature neuroscience* 2019; **22**(3)**:** 343-352.
