# Supplementary material for: Genome-wide association study implicates the role of TBXAS1 in the pathogenesis of depressive symptoms among the Korean population
Source: Transl Psychiatry. 2024 Feb 6;14:80. doi: 10.1038/s41398-024-02777-3 (PMC10847124; doi:10.1038/s41398-024-02777-3)

**Supplementary Figures**

**Figure S1**. Workflow of quality control steps.

^
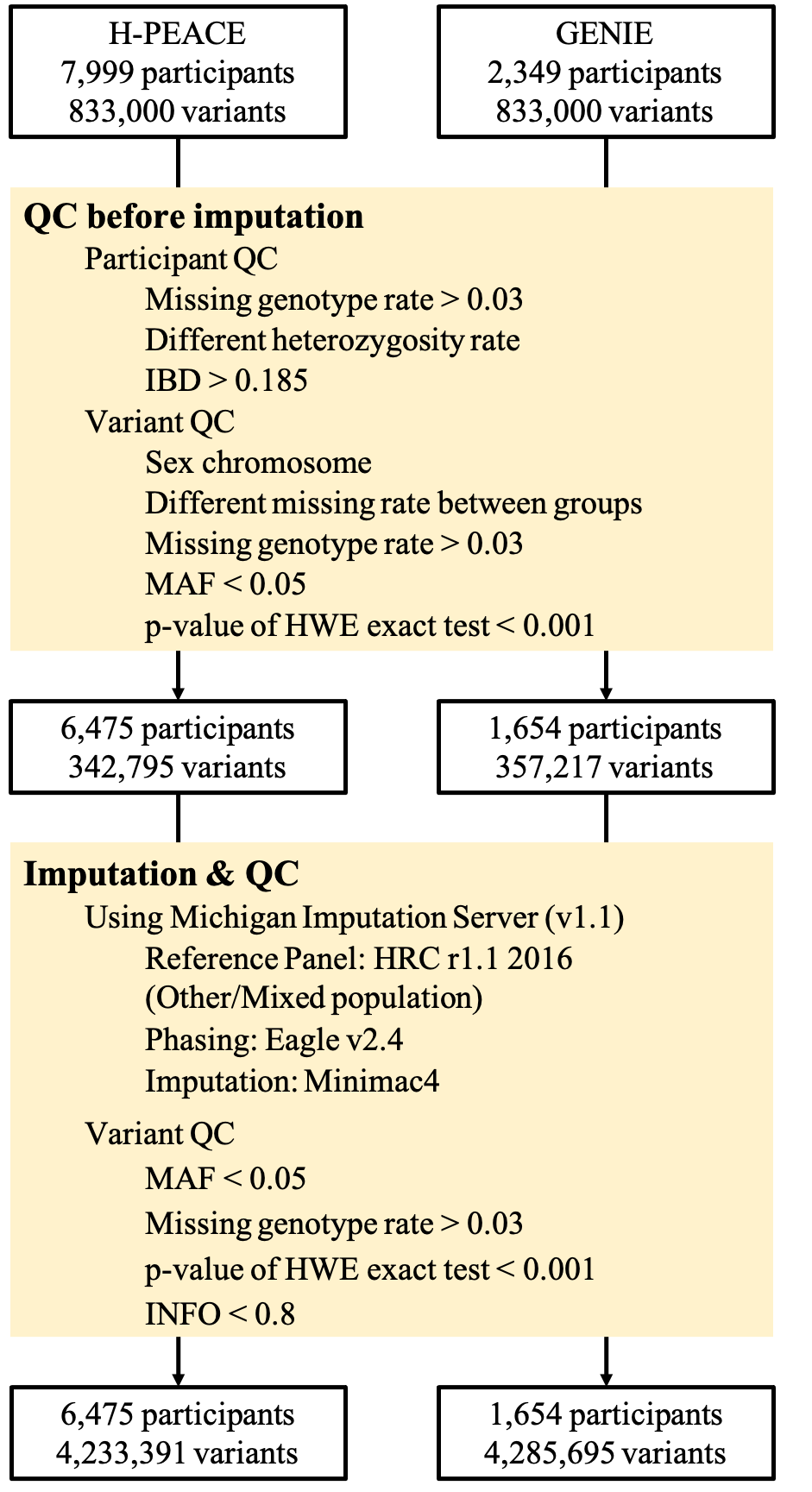
^

**Figure S2**. GWAS results of each cohort. (A) Manhattan plot and (B) quantile-quantile (qq) plot in H-PEACE cohort. (C) Manhattan plot and (D) qq plot in GENIE cohort. Blue line in Manhattan plots represents $1.00\times{10}^{-5}$ significance level and grey regions in qqplots are 95% confidence interval of *P*-values. p, *P*-value; GIF, genomic inflation factor.

^
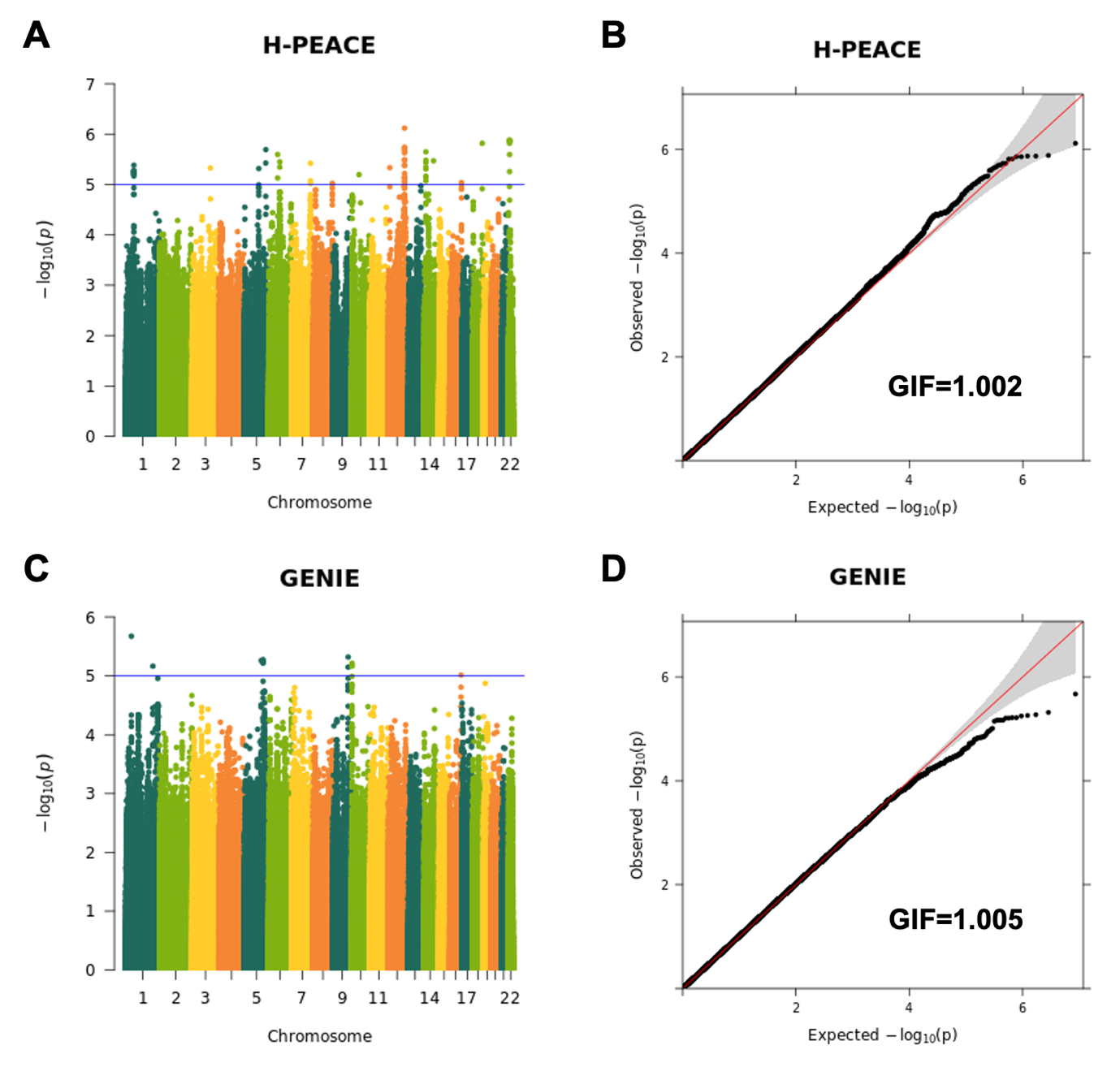
^

**Figure S3.** Violin plots of predicted gene expression levels. *LSS* expression levels of participants in (A) H-PEACE and (B) GENIE cohorts are represented according to their phenotype. (C) and (D) shows those of *YBEY* in H-PEACE and GENIE, respectively. $\beta$, estimate of expression difference between case and control; *p*, *P*-value; FDR, false discovery rate.


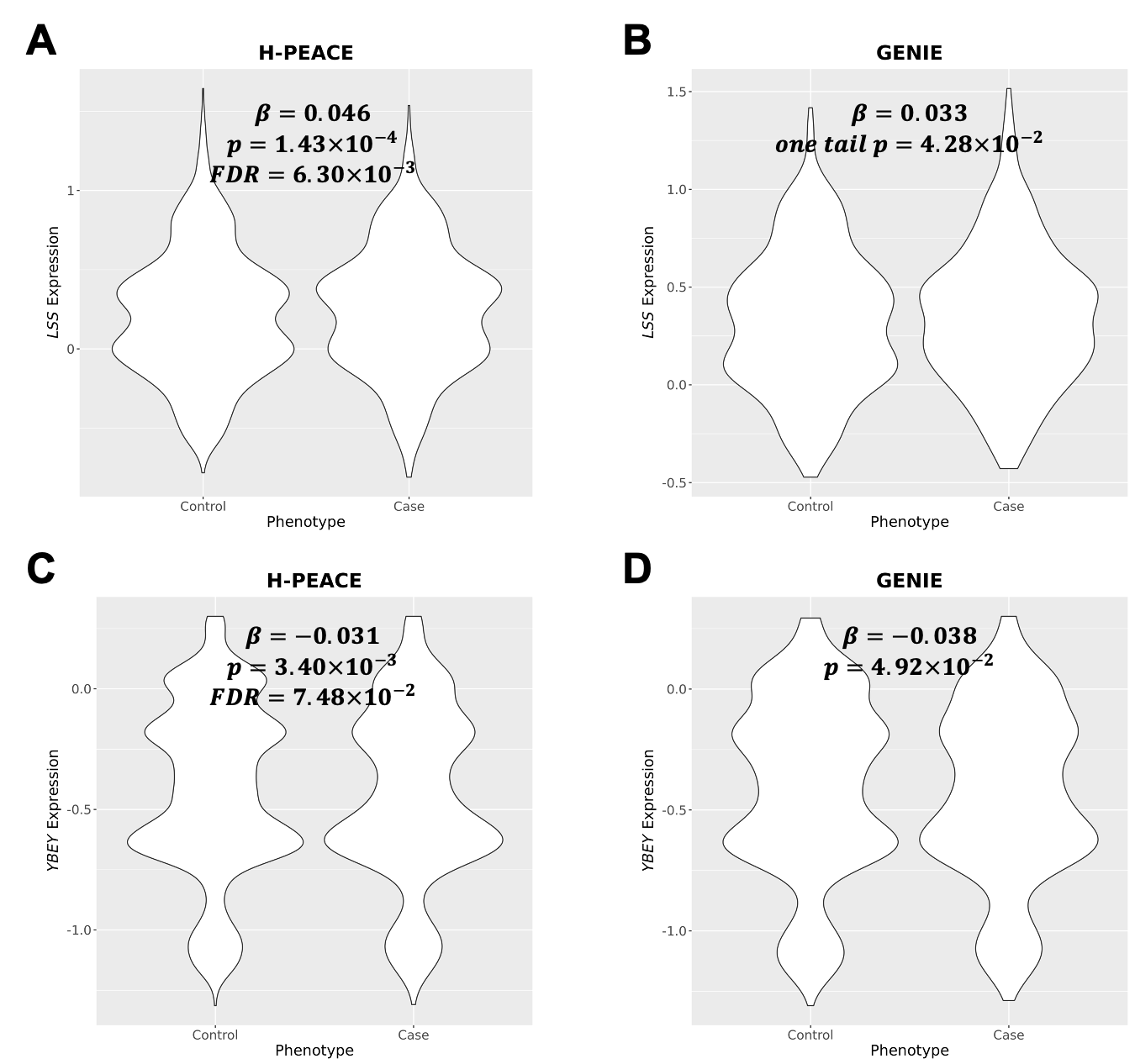


**Figure S4.** Results of Mendelian randomization analyses. Causal effects to risk of depressive symptoms of (A) *TBXAS1* and (B) *LSS* with instrument variables (IVs) selected at $1.00\times{10}^{-5}$ significance level, respectively, and (C) *YBEY* with IVs extracted at $5.00\times{10}^{-8}$. Dashed lines represent regression lines and yellow dots and lines are effects of IVs and their $\pm1$ standard error, respectively. $\beta$, estimate of casual effect; p, *P*-value.

**
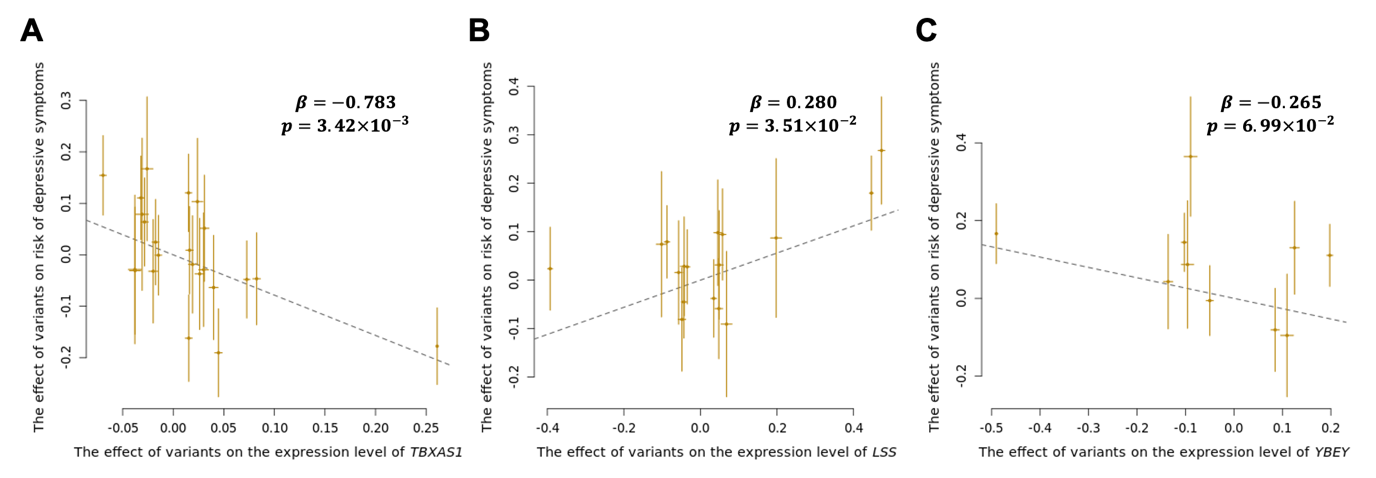
**

**Figure S5.** Item correlation. (A) Plot representing pairwise correlation of scores between items. Upper and lower triangle shows the correlation in H-PEACE and GENIE cohorts, respectively. Estimates of correlation are shown in colored circle ranging from red to blue and size of the circle means significance of correlation expressed as $-\log_{10} p$ where 30 was set to the upper limit. If *P*-values were larger than 0.05/210, circle were not drawn. (B) Cluster dendrogram of items of combined participants of the two cohorts. It was drawn based on Pearson correlation of scores of each item. (C) Plot of pairwise genetic correlation of items. Upper and lower triangle represents estimate of the correlation in H-PEACE and GENIE cohort, respectively. Degree and significance of the correlation were shown by color and size, respectively. Upper bound of $-\log_{10} p$ was set to 4. Only estimable correlations were represented as circle. p, *P*-value.


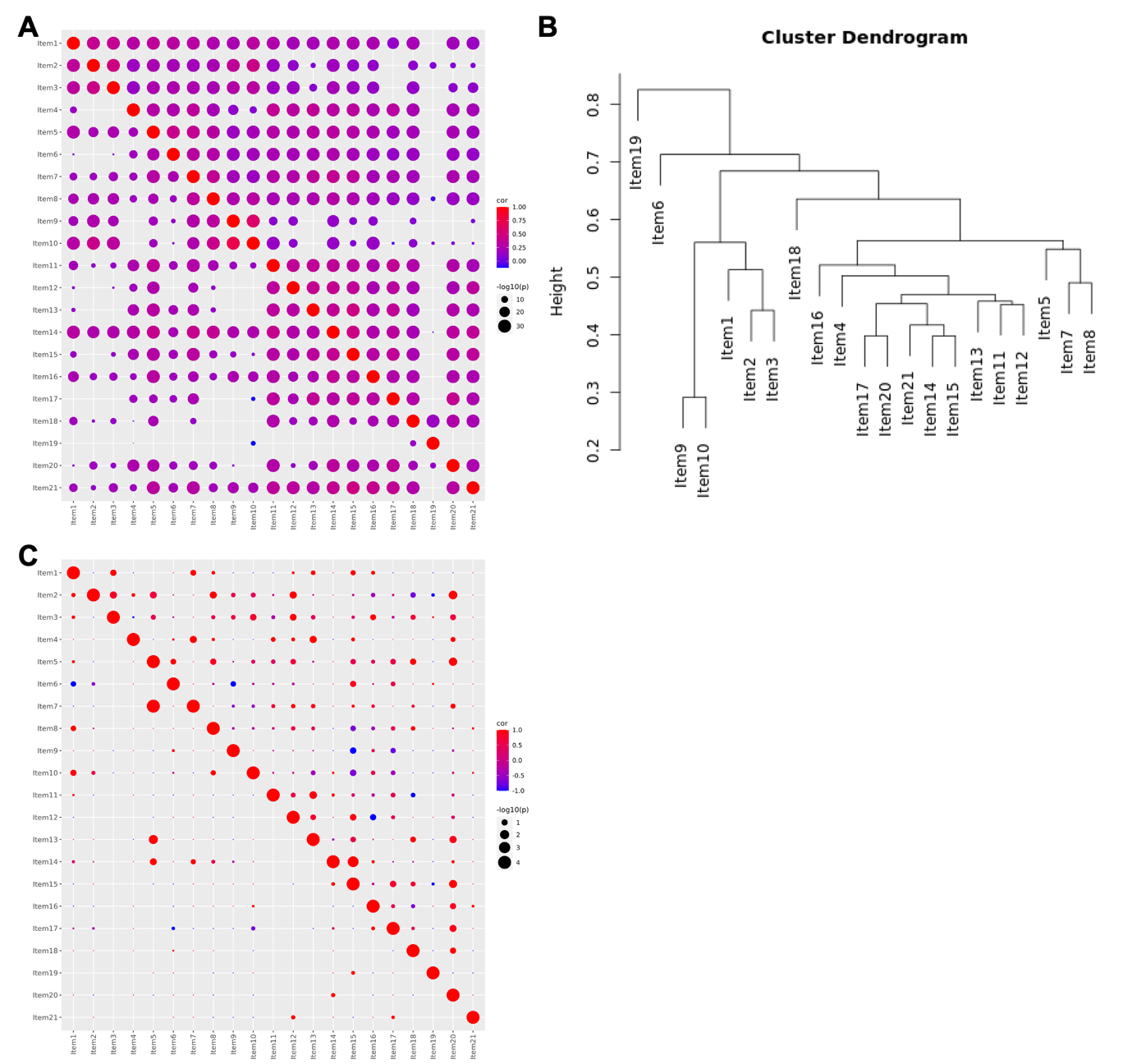


**Figure S6.** Manhattan plots of each cluster. (A)-(D) represent GWAS results of cluster 1-4, respectively. Red and blue lines represent significance level $5.00\times{10}^{-8}$ and $1.00\times{10}^{-5}$, respectively. p, *P*-value.


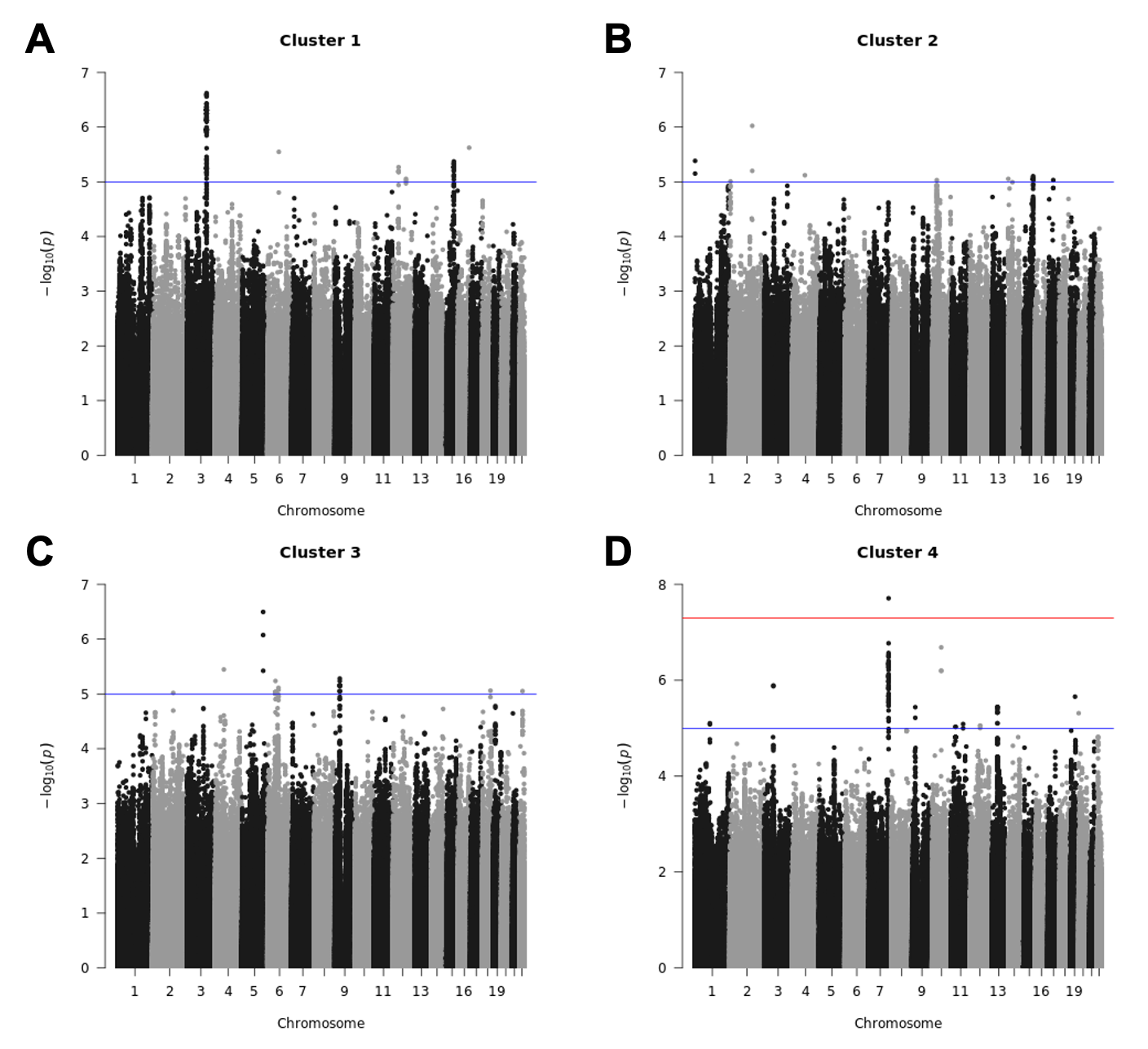


**Figure S7.** Manhattan plots of each item. (A)-(U) represent GWAS results of item 1-21, respectively. Red and blue lines represent significance level $5.00\times{10}^{-8}$ and $1.00\times{10}^{-5}$, respectively. p, *P*-value.


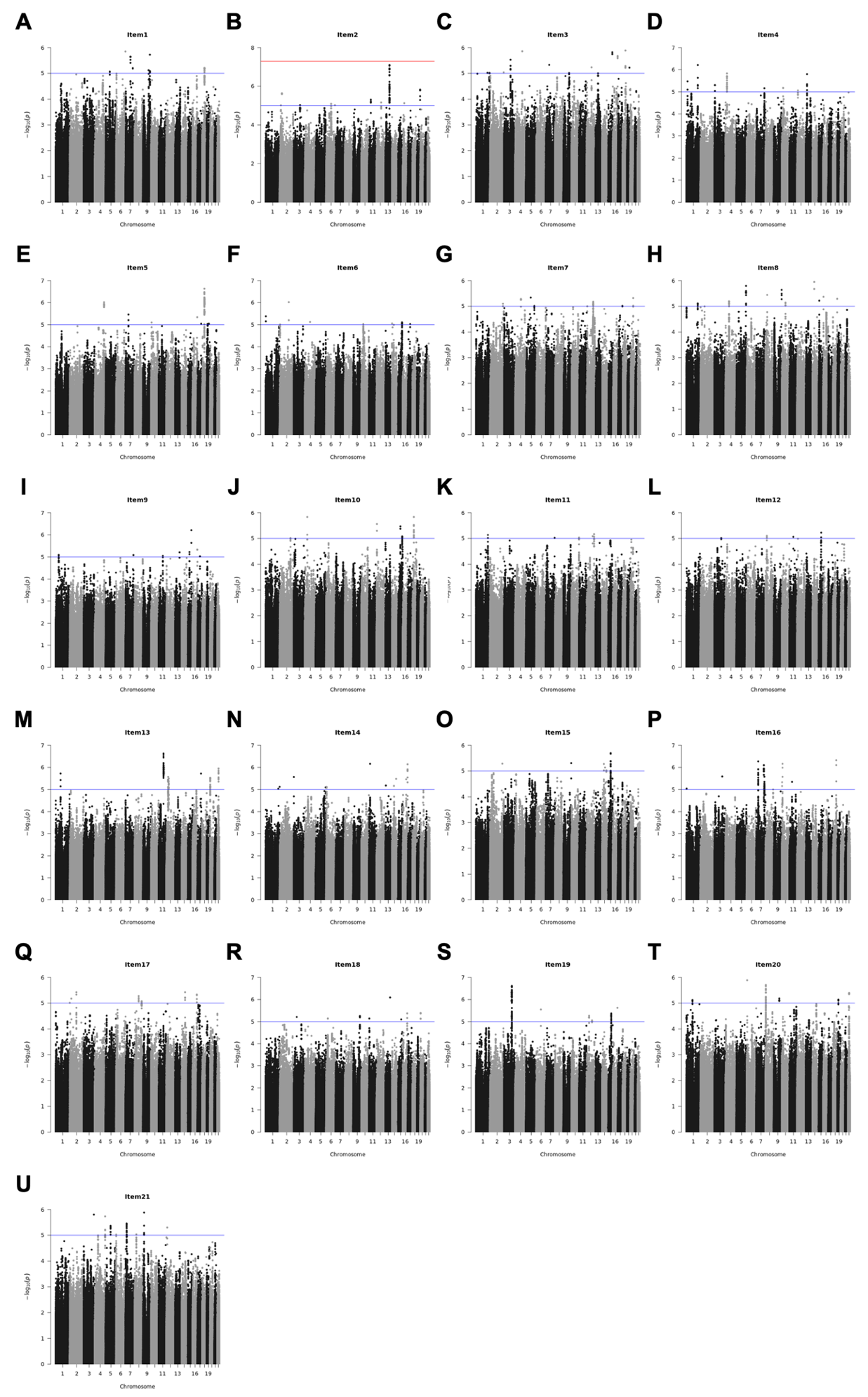

Supplement: Supplementary file 2 — Supplementary Figures [file 41398_2024_2777_MOESM2_ESM.docx]
